# Ethnoveterinary Medicine and Ethnopharmacology in the Main Transhumance Areas of Castilla-La Mancha (Spain)

**DOI:** 10.3389/fvets.2022.866132

**Published:** 2022-05-03

**Authors:** Diego Rivera, Alonso Verde, José Fajardo Rodríguez, Segundo Ríos, Francisco Alcaraz, Carlos Cárceles, Juana Ortíz, Arturo Valdés, Jose Reyes Ruíz-Gallardo, Aida García-Flores, José Antonio Palazón, Concepción Obón

**Affiliations:** ^1^Departamento de Biología Vegetal, Facultad de Biología, Universidad de Murcia, Murcia, Spain; ^2^Grupo de Investigación en Botánica, Etnobiología y Educación, Laboratorio de Sistemática y Etnobotánica, Instituto Botánico, Universidad de Castilla-La Mancha (UCLM), Jardín Botánico de Castilla-La Mancha, Albacete, Spain; ^3^Biological Research Station-Botanical Garden of Torretes, Institute of Biodiversity CIBIO, University of Alicante, Alicante, Spain; ^4^Departamento de Farmacología, Facultad de Veterinaria, Universidad de Murcia, Murcia, Spain; ^5^Departamento de Sanidad Animal, Facultad de Veterinaria, Universidad de Murcia, Murcia, Spain; ^6^Departamento de Ecología e Hidrología, Facultad de Biología, Universidad de Murcia, Murcia, Spain; ^7^Centro de Investigación e Innovación Agroalimentaria y Agroambiental (CIAGRO), Escuela Politécnica Superior de Orihuela (EPSO), Universidad Miguel Hernández de Elche, Orihuela, Spain

**Keywords:** ethnobotany, Iberian Peninsula, medicinal plants, transhumance, poisonous and harmful plants, ichthyotoxic, poisonous animals

## Abstract

In this study, we document the practices of ethnoveterinary medicine and ethnopharmacology in the context of traditional transhumance routes that cross Castilla La Mancha from north to south. Transhumance is a type of grazing system that allows advantage to be taken of winter pastures (wintering places) and summer pastures by seasonal movement, twice a year, of cattle and their shepherds. Our study is based on over 200 interviews (from 1994 to 2021) conducted in 86 localities along eight major transhumance routes “cañadas reales” and 25 other minor transhumance routes, and involved 210 informants, 89 single and 121 groups, and 562 individuals, of which the majority were men. Sixty-three recorded pathologies and their treatments are discussed. Two hundred and two species and substances, belonging to 92 different families, have been recorded from the interviews, of which most are plants. Amid the toxic plant species, the most cited in the interviews are *Erophaca baetica* (L.) Boiss., *Lupinus angustifoliu*s L., and *Oenanthe crocata* L. Some of the species reported as toxic were reservoirs of pathogens or markers for dangerous areas. One of the fields most widely covered in our study is that of prevention, protection, and control of endo- and ectoparasites. This control is carried out mainly by means of aromatic plants. As a polyvalent species, *Daphne gnidium* L. is outstanding, and it contributes one-tenth of the records of our study. Among the species of fundamentally therapeutic use, *Cistus ladanifer* L. stands out by far. Principal Coordinate Analysis (PCoA) based on the repertories of ingredients, separates the routes whose most important sections run through siliceous terrain with its characteristic flora, especially in the provinces of Ciudad Real and Toledo, from the routes that run through the limestone terrain of Albacete and Cuenca, and link the Eastern Mancha and the “Serranía de Cuenca” with Andalusia and the Spanish Levant.

## Introduction

The way in which La Mancha shepherds dealt with the health problems of livestock, especially transhumant livestock, is a subject that involves the use of natural resources and the traditional elaboration of remedies based especially on herbs, as well as an empirical knowledge of various pathologies. Wolves follow transhumant herds along the trails, taking advantage of the situation to attack stragglers, old or sick animals, which become easy prey when separated from a herd (1). In addition to vermin attacks, transhumant livestock are subject to damage from long journeys, blows and wounds, various infectious diseases, parasites, insect and scorpion stings, snake bites, and poisoning from consumption of toxic plants. Added to this are problems of childbirth and lactation and other problems that herders have to face with the meager resources available to them. Pastoralists had to have good knowledge of these local resources, of poisonous plants and animals, and even of places to avoid, for example “cursed fields” where anthrax persisted (2).

One of the newest directions in ethnobiology, ethnoveterinary research and development (ERD), is no more than 50 years. Ethnoveterinary is a term coined by McCorkle (3) and defined as “the systematic investigation and application of popular veterinary knowledge, theory, and practice.” In parallel, veterinary is recovering the use of medicinal plants in different contexts (4, 5).

In this study we approach ethnoveterinary medicine and ethnopharmacology in the context of traditional transhumance routes that cross Castilla-La Mancha from north to south.

From Latin *humus*, earth, from which the word human also comes, the one who inhabits the earth, and *trans* (beyond), transhumance means to move from land to land. It is a type of grazing system that allows advantage to be taken of the winter pastures (wintering places) and summer pastures by seasonal movement, twice a year, of cattle and their shepherds. The fact of moving cattle and shepherds, long distances and for months, supposes notable conditioning factors both in terms of affected cattle, prevalent diseases, and available resources.

These movements were very frequent throughout Spain and in other Mediterranean countries because of the diversity of their climates and differences between mountain pastures and those offered by warmer, coastal, or lower-altitude areas. There were two types of transhumance: long transhumance and short transhumance, which took advantage of altitudinal variations in pastures located at close distances (6).

The movement of cattle from one place to another, with the movement of numerous animals over long distances, made it necessary to regulate their passage and create and protect livestock routes that would facilitate their movements, with sufficient width (“cañadas reales” 75 m wide, “cordeles” 37.5 m, and “veredas” 20 m), to allow for the feeding of cattle during their journey, availability of areas such as resting places and shelters, and access to necessary troughs and lagoons (2, 7).

The institution of “La Mesta” (created by King Alfonso X in 1273) was key to the organization of these livestock routes. Of the “cuadrillas” or groups of shepherds with which the Mesta was organized, one of them was Cuadrilla de Cuenca, in what is now Castilla-La Mancha (8).

Shepherds were organized into groups led by a mayor who organized the transfer (9). When one route was followed for the first time, the travel was planned in advance with great care in detail: the route was recognized, the resting places were chosen, the pertinent permits were obtained for the use of pastures, and the documentation was kept that opened the way for them and exempted them from taxes (2).

At the end of the twentieth century, transhumance became testimonial because of the decrease in extensive livestock farming and massive conversion to stabled livestock, associated with the generalized use of feed and fodder. Transport of livestock by trucks reduced traditional routes to only two extremes: summer pastures and winter pastures. However, today there are still some herders who practice transhumance more to follow tradition than for practical reasons.

In Castilla-La Mancha, summer pastures are located in highest and coldest mountain areas of the region, situated in the Central and Iberian systems, such as the Serranía Alta de Cuenca and the Montes Universales. Wintering is restricted to warmer areas such as the Alcudia Valley (Ciudad Real), and often across borders in neighboring regions, at much lower altitudes or south-facing valleys, as in Andalusia (Sierra Morena or Los Pedroches), or near the Mediterranean coast, as in the regions of Murcia or Valencia. These are connected by a complex network of transhumance routes (10).

We can find sparse ethnoveterinary information in studies on Castilla-La Mancha or some of its areas (2, 7, 11) and the series of publications on transhumance called “Cuadernos de la trashumancia,” published by ICONA, in the Alcudia Valley (12), Albarracín-Cuenca-Molina (13), Sierras de Alcaraz, Cazorla and Segura (14), and Campos de Calatrava-Montiel (15).

Most of the ethno-veterinary research on Spain is widely dispersed in numerous works, generally published in Spanish, frequently within ethnobotanical publications of a general nature and referring to specific territories. This makes access difficult, especially for non-Spanish-speaking researchers. General ethnoveterinary studies cover part of the following main areas: southern: Andalusia (16, 17) and Extremadura (18–22); northern: Aragón (23–27), Basque Country (28–31), Galicia (32, 33), and Navarra (34, 35); eastern: Balearic Islands (36) and Catalonia [(36, 37)]; Central: Castilla-La Mancha (38, 39), Castilla-León (40–42), and Canary Islands (43).

Several specialized studies cover the ensemble of Spain, such as those by González et al. (44–46), González and Vallejo (47), and González et al. (48) studied plant remedies used in the Iberian Peninsula to treat wolf bites in cattle.

In Castilla-La Mancha, various general studies on ethnobotany have been carried out where the traditional uses of medicinal plants are dealt with including ethnoveterinary information (49–56). Ethnoveterinary information can be found in ethnopharmacology studies on Castilla-La Mancha (49, 57–62). Bremner et al. (63) analyzed several plants of the SE of Spain for their potential use as anti-inflammatory.

Although the published studies mentioned above collect ethnoveterinary data, they are very partial, as they are either extremely local or very specialized, or excessively superficial. There is a lack of a broad overview describing specific pathologies and remedies, including their formulations and modes of preparation and administration, in the territories of Castilla-La Mancha traversed by the transhumance routes, and the local resources used.

The specific aims of this research are:

- To document the pathologies, formulation, and uses, particularly identification of species of animals treated, and related knowledge of transhumance areas of Castilla-La Mancha. For this, we intend to identify the different ingredients and their relative importance in the whole, as well as determine types and styles and their geographic patterns if these exist.
- We also intend to analyze the knowledge of toxic organisms, plants, animals, and fungi for livestock, both those that are toxic *per se* and those that could act as reservoirs of pathogens. In this context, we will investigate the use of ichthyotoxic species.

## Materials and Methods

### Study Area

**Figure 1:**
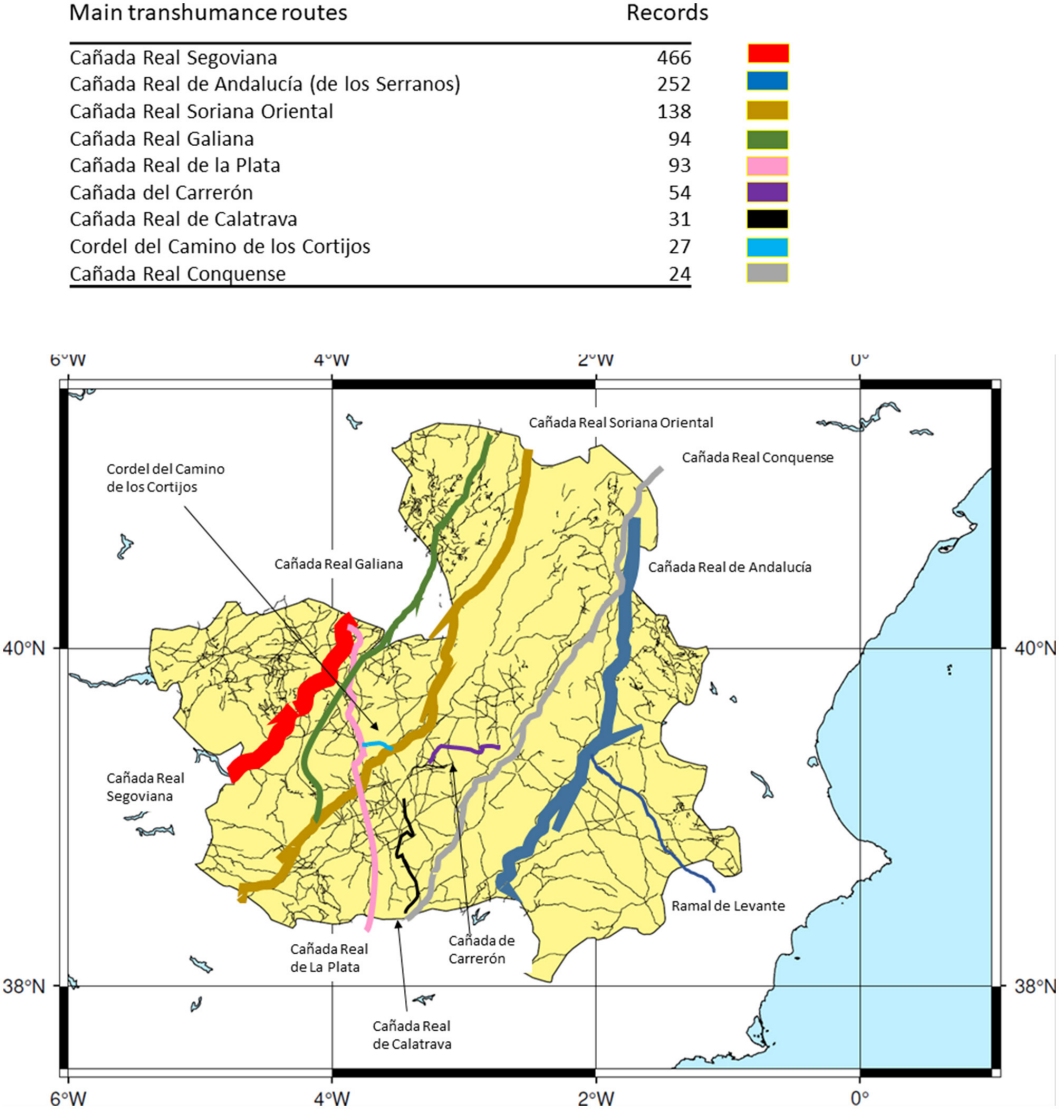
Location map of the studied transhumance routes in Castilla-La Mancha. Note: the width of each one of the routes is proportional to the number of records linked to this particular route. Map: F. Alcaraz.

This article focuses on the study of the areas covered by the most important cattle transhumance routes in Castilla-La Mancha, especially in the provinces of Albacete, Ciudad Real, Cuenca, and Toledo (Figure 1).

Our ethnoveterinary and ethnopharmacological research concentrates in three major areas: The Serranía de Cuenca, the province of Albacete and areas neighboring the Upper Guadiana River and its tributaries (Azuer, Cigüela, Estena, and Záncara River), especially in two national parks, those of Cabañeros and Las Tablas de Daimiel, and the natural park of Lagunas de Ruidera in Ciudad Real province (Spain) and close localities of Albacete, Cuenca, and Toledo provinces (Figure 1).

We draw maps using GMT (64).

### Data Collection and Plant Specimens Identification

**Figure 2:**
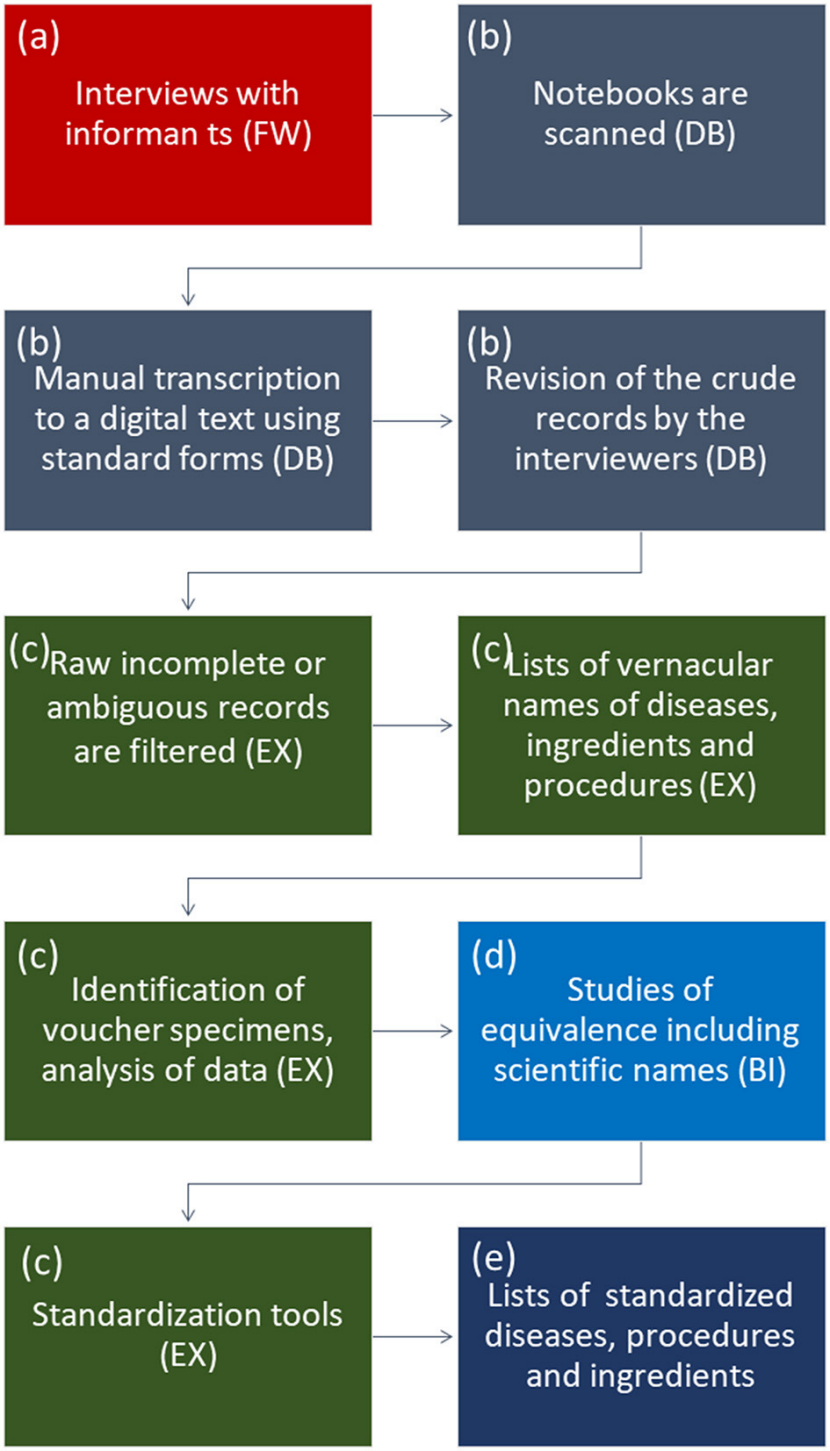
Sequence followed for determining/interpreting ingredients and pathologies. Color codes: **(a)** (red) field work, **(b)** (gray) database, **(c)** (green) Excel book, **(d)** (light blue) bibliographical analysis, and **(e)** (deep blue) final report as Supplementary Table 1.

We followed a logical sequence for recording, processing, and standardizing data, which is summarized in Figure 2.

In our methodology, we distinguish between interviews and informants. Interviews are conducted on a specific date, and the person or persons interviewed constitute the informant. This implies that the informant can be an individual or a combination of individuals and that they can be interviewed one or more times. In the case of a group informants (couples, families, etc.), they are only considered to be the same informant if the composition of the group is maintained in different interviews. Records are attributed to the informants, although information on the specific interview in which the data were collected is available in the database. Frontiers rules preclude identifying informants or indicating their age or gender.

**Figure 3:**
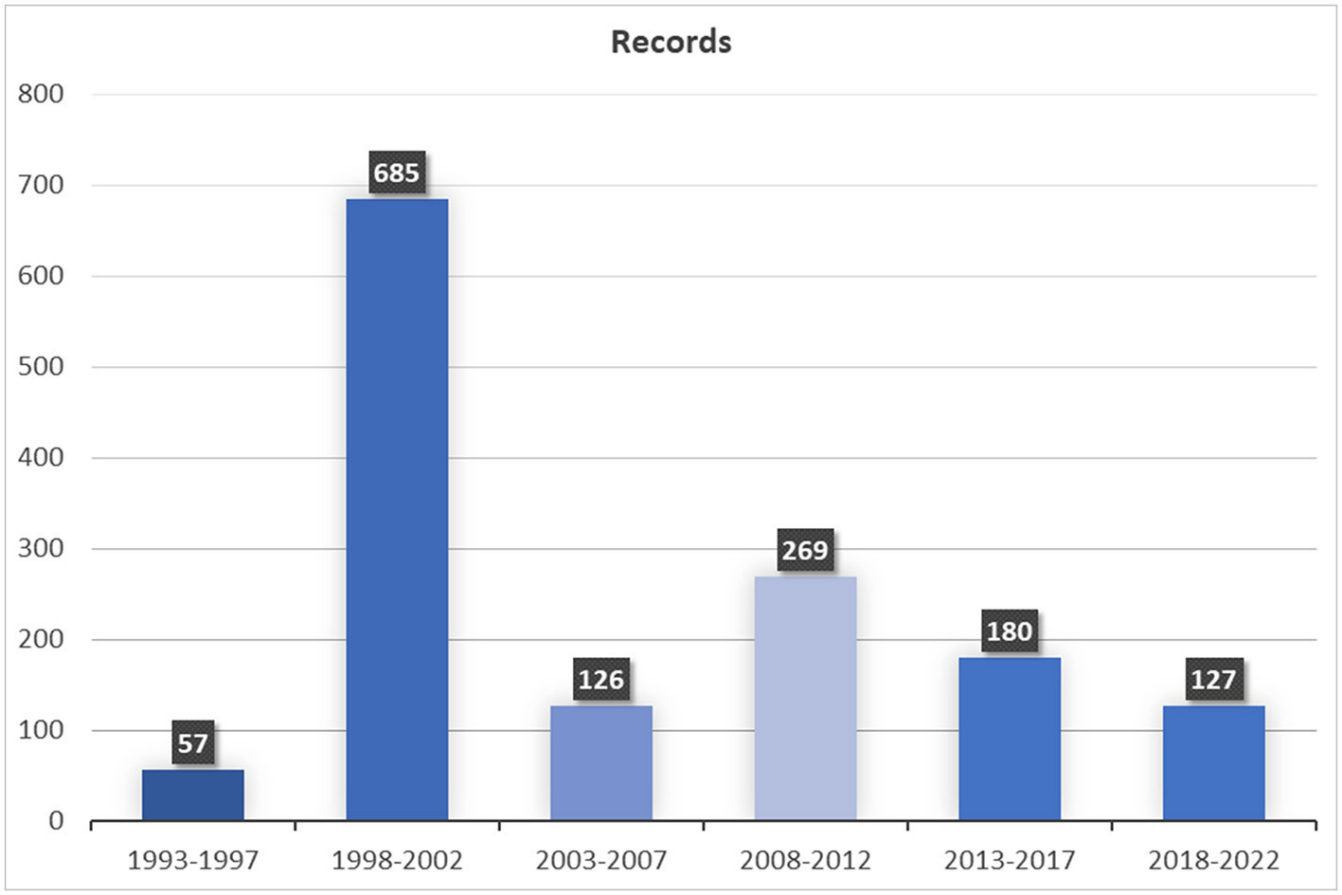
Chronology of the field work.

Our study is based on 237 interviews, individual or with groups of people. The number of individuals who made up the informant groups ranged from 2 to 30. The field work took place from 1994 to 2021 (Figure 3) and comprised 86 localities along eight major transhumance routes “cañadas reales” and 25 other minor transhumance ways (Figure 1).

The 237 interviews involved 210 informants, 89 single, and 121 groups, of which 88 were independent (neither interviewed simultaneously nor belonged to the same family). Taking into account the number of individuals in the informant groups and the persons interviewed individually, the list of different persons interviewed amounts to 562. The majority of the individuals interviewed were men (405), while women were only 157, and numerous records were obtained by interviewing 29 couples and families. Finally, some records were obtained from interviews with groups of four to 30 people.

In all the cases, the interviewees were informed of the purpose of the study, and informed consent was obtained. ISE protocols (65) were followed. We must mention here that the interviews carried out generally covered a wide range of ethnobiological topics, of which, in this study, only records of veterinary interest are analyzed.

We tried to cover the areas linked to the major transhumance routes as continuously as possible throughout the investigation period, with some interruptions. The discontinuities are, unfortunately, often due to the death of our informants, given their ages.

We collected voucher-specimens of plants and fungi, which were deposited in the herbarium ALBA (Universidad de Castilla-La Mancha, Albacete, Spain) as a rule, except for the older samples that were deposited in MUB (Universidad de Murcia, Murcia, Spain). Further details on these herbaria are available at NYBG (66). For animals, we took photographs that are available at the repository of images (http://ethnobiowetlands.es/etnofauna/) of the “Etnobiología de los humedales” portal. The identity of plant ingredients was determined based on the collected specimens and with the use of existing standard regional floras, and the names were verified with the botanical literature available at BHL (67), monographs, and databases, Anthos (68), Euromed (69), Tropicos (70), GRIN (71), IPNI (72), NCBI (73), Fitoterapia.net (74), and most importantly, nomenclature was standardized following the procedures suggested by Rivera et al. (75), according to Plants of the World (76), which replaced TPL (77) that is no longer updated. For *Salvia* nomenclature, we followed Reales et al. (78), and for *Sideritis* (79).

The ingredients of zoological and mineral origin appear, with different relevance, in almost all the localities studied. The animals were identified in the field with our informants, according to the documents of the Fauna Ibérica project (80), but no samples were taken because of the capture restrictions imposed by Spanish legislation, and because, lately, the use of animals is decreasing. We favored photography instead of capturing animals.

The mineral ingredients were identified with the help of García-Guinea and Martínez (81) and medicinal waters with Maraver (82). We updated the nomenclature of minerals according to the New International Mineralogical Association List of Minerals (83, 84) and of organic and inorganic chemicals following Pubchem (85). The nomenclature of the animal species treated and ingredients of zoological origin was updated using the Global Biodiversity Information Facility (86) and García and Gisbert (87). The nomenclature of bacteria followed LPSN (88).

The scientific names of biological species, with authorities, are listed in alphabetical order of families, genera, and species in Supplementary Tables 1, 2. For all species, the standardized acronyms of the authors have been added to the scientific names in the main text, when they appeared for the first time.

The identification of livestock pathologies is based on the explanations of our informants, experience of the veterinarians in the research team, and collaboration of local veterinarians. The following classic *albeiteria* and veterinary studies and manuals have also been extraordinarily useful: Daubenton and González (89), Cubillo (90), Sánchez (91), West (92), González-Stuart (93), Xie et al. (4), Boden (94), and Merck Manual (95).

### Data Management and Analysis

#### Data Organization and Preliminary Processing

The information collected from the informants during the interviews was introduced into a database. Both database design and specific software have been created to manage the ethnobiological information documented in the National Parks of Cabañeros and Tablas de Daimiel, and neighboring areas of the upper Guadiana River.

Firebird has been used as a Database Manager System (96), which is Open Source, and therefore of free distribution. The software has been created using Delphi^®^ 10.2 Tokyo Professional (97). From this database, an Excel-compatible output was generated.

Regarding the inclusion criteria for the medicinal recipes, we included the recipes addressed to the prevention or treatment of recognizable pathologies of livestock and domestic animals whose equivalences are defined in the general veterinary literature. We included, therefore, veterinary formulas and references to toxic plants, poisonous animals, and mineral substances of known toxicity to humans and livestock. General dietary prescriptions not related to specific pathologies were excluded here.

To interpret the vernacular names of pathologies, Cubillo (90), Sánchez (91), West (92), Morcillo et al. (2), and DRAE (98) were consulted. The Merck Manual (95) has been used to determine the groups of pathologies.

All ingredients of the analysis were identified and classified in terms of their main biogeographical profiles and zones of origin according to the next main categories: Widespread, Mediterranean, Europe, Spain, Asia, America, Pacific islands, and Northern and Eastern Hemisphere, following Rivera et al. (99) and GBIF (86).

In order to standardize the vernacular nomenclature, especially for vernacular names of diseases and of ingredients of medicines, some dictionaries/thesauri were created in Excel, which were then applied to the automatic generation of the final text.

#### Data Analysis

In order to determine how different these 31 transhumance “cañadas” are, considering as a reference the 202 species documented (see Section Medicinal and toxic species, ingredients, and formulations of Results), we calculated the pairwise differences between samples in the form of a dissimilarity matrix. However, we excluded species recorded in one single locality and “cañadas” with <4 ingredients. Thus, the filtered crude matrix was reduced to 30“cañadas” and 102 species. Data were considered for each route over the whole period of 1994–2021. We adopted this approach, since splitting into shorter periods produced unmanageable too small subsamples.

The crude matrix of presence/absence of ingredients was used to compute a dissimilarity matrix using Darwin 6 V.6.0.9 (100, 101). The Sokal-Sneath dissimilarity index was calculated (un2) [1].


(1) $$\begin{array}{r} d_{\text{ij}}=2\left(b+c\right)/\left(a+2\left(b+c\right)\right) \end{array}$$

where d_ij_ is the dissimilarity between samples i and j; a: number of variables where x_i_ = presence and x_j_ = presence, b: number of variables where x_i_ = presence and x_j_ = absence, and c: number of variables where x_i_ = absence and x_j_ = absence. Dissimilarities are even and are Euclidean distances. The dissimilarity is = 0 for two samples sharing the 202 species and = 1 for two samples that present 0 species shared. This index concerns “presence/absence” data where only the “presence” modality is informative, and modality “absence” expressing mainly an absence of information. These two modalities are not symmetrical, and their exchange leads to a completely different dissimilarity value. This index considers that a common absence for two units is uninformative to measure their dissimilarity (101). Therefore, here, similarity reflects the number of coinciding species, and dissimilarity is inversely proportional to this.

Pairwise dissimilarities can be represented in a multidimensional space, but in order to obtain a meaningful graphic representation of these relationships in a two-dimensional plane, we conducted cluster analysis (102).

We used the agglomerative hierarchical method that arranges clusters into a hierarchy so that the relationships between different groups are apparent. Minimum variance clustering (Ward's method) focuses on determining how much variation is in each cluster. In this way, clusters will tend to be as distinct as possible, since the criterion for clustering is to have the least amount of variation (102).

Principal coordinates analysis (PCoA) is a member of the factorial analysis family working on distance matrices. It considers the space of high dimension defined by the distances between units two by two. This space has too high dimension to be readable, so PCoA searches for a subspace of low dimension where distances between units are as close as possible to originate distances. Working with the above dissimilarity matrix, we represent the units against the first and second axes. Details of the PCoA procedure are described in Perrier and Jacquemoud-Collet (101).

## Results and Discussion

### General Results

The knowledge recorded during the interviews with our informants is very variable: the number of ethnoveterinary records per informant ranges from 1 to 73 [with an average (av.) of 6.9 and standard deviation (sd.) 9.05]. By record, we mean each chain of data comprising species and part used (both constitute an ingredient): formulation, form of preparation, form of administration, animal treated, and therapeutic indication.

The ingredients mentioned per informant varied from 1 to 29 (av. 4.6, sd. 4.17), and the pathologies mentioned ranged from 1 to 21 (av. 3.5, sd. 2.92). In general, the ethnoveterinary information collected was scarce: more than 60% of the informants provided only 1–5 records, while only 4.3% provided more than 20 records. Similarly, ~45% mentioned 1–5 ingredients and only 1.4% more than 20 ingredients.

In parallel, more than 85% of the informants described 1–5 pathologies, whereas only c. 3% described remedies for more than 10 pathologies. It should be noted here that we quantified the knowledge that the informants were willing to share with us, and that we were able to record through the semi-structured interviews but not their actual knowledge. This applies to most ethnobiological studies.

It is appropriate here to recall that our interviews covered a wide range of subjects; therefore, we also usually recorded information on human pathologies, agricultural diversity and practices, and food and crafts, together with specific veterinary recipes and knowledge on poisons. We did not proceed to monographic interviews with our informants on health issues. Interview sessions lasted between half and 3 h, and key informants were repeatedly interviewed.

The low proportion of women interviewed (c. 30%) is related not to our sampling strategy but to the difficulty of finding in this area women who are willing to be interviewed, and we must remember that although nowadays entire families participate in transhumance, the work of shepherding has been essentially a male occupation (2). Interestingly, the portion of women interviewed who talk about ethnoveterinary aspects is higher than that of ones who are willing to talk about traditional medicine (62), and overall, their level of knowledge is similar to that of men. This is particularly remarkable for the Alto Guadiana area, where this could be interpreted in terms of the role played locally by women as healers (103) and the tradition of hiding this knowledge. For instance, “Las Curanderas” (the female healers) is the official name for a place and a local road in Daimiel (104) in the vicinity of Puente Navarro. Women healers still lived in this area in 2018, but they were only willing to talk about the ornamental plants in their home gardens, and mostly avoided talking to us about their healing practices, which focused mainly on humans but also on livestock (62).

With regard to gender and the amount of information recorded in terms of, respectively, the records and ingredients mentioned, it was similar although slightly higher in men, av. 6.5 records (sd. 7.38) and av. 4.4 ingredients (sd. 3.8), with a maximum of 79 and 29, respectively, from a single informant. In women, it was av. 6.4 records (sd. 3) and av. 4.1 ingredients (sd. 1.6), with a maximum of 20 records and 13 ingredients. It should be noted that when the interviews included men and women simultaneously, often husband and wife, the results were slightly better than in single interviews (7.7 records and 5.1 ingredients on average, s.d. 4.26 and 2.41 respectively) with a maximum of 29 records and 20 ingredients. Many of our informants worked simultaneously as farmers and herders, with a greater dedication to one or the other depending on the season or through a rational distribution of tasks within the family.

### Species and Pathologies

#### Species Treated

One topic that is not recorded in the interviews but has been repeatedly observed in fields by members of the research team is that not all types of livestock were cared for equally. The greatest care was for equids (horses, mules and donkeys), then animals for breeding and milking, which received the best food and care, and then there are animals that, when injured, were not treated but rather were sacrificed to take advantage of their meat, and the same happens with females that do not breed, and this correlates with the differences in number of records.

Our informants have used the term “ganado,” livestock category most frequently (597 records) when they described non-specific pathologies that affect various groups of domestic animals, especially those involved in the process of transhumance (Supplementary Table 1). Second, especially when referring to poisonous plants and animals, they indicate that they can affect animals and humans (290 records).

Among equids, mule diseases prevail (*Equus asinus* Linnaeus, 1758 x *E. caballus* Linnaeus, 1758, 82 records), followed by horses (*Equus caballus* Linnaeus, 1758, 49 records), and donkeys (*Equus asinus* Linnaeus, 1758, 15 records).

Diseases of sheep in general and especially ewe (*Ovis aries* Linnaeus, 1758, 71 records), together with those of lambs (63 records) and rams (three records), constitute another large group among those registered.

Diseases of goats (*Capra hircus* Linnaeus, 1758, 78 records) and knowledge of plants that are toxic to them also provide a considerable body of data.

Bovine diseases, especially *Bos taurus* Linnaeus, 1758, in general (six records) calves (3) and cows (35 records), constitute the least represented group.

Dogs (*Canis familiaris* Linnaeus, 1758), despite their importance in herding and handling cattle, have only contributed 15 records.

Although it is not the priority topic of the study, references have incidentally been collected to diseases of farmyard animals such as pigs (*Sus domesticus* Erxleben, 1777, 22 records), rabbits [*Oryctolagus cuniculus* (Linnaeus, 1758), seven records], chickens [*Gallus Earleen* (Linnaeus, 1758), 17], pigeons (*Columba livia* Gmelin, 1789, 1), and even sparrows [*Passer domesticus* (Linnaeus, 1758), 1].

Disinfection of hives (*Apis mellifera* Linnaeus, 1758) was mentioned in 17 records.

Freshwater edible fish (65 records) are mentioned as victims of ichthyotoxic plants.

#### Pathologies Identified

The 63 recorded pathologies are listed below, organized by major groups according to the criteria of the Merck Manual (95) in the order of decreasing percentage of records.

Concerning emergency medicine and critical care (18.8% of records), the three categories recorded include disorders of the hoof, emergency hoof care, and, especially, wound healing (16% of records) especially “mataduras.” “Mataduras” are sores that a horse or a mule gets from tack or from rubbing of an implement, or from a saddle being wet with sweat (105).

For the integumentary system (including ectoparasites; 12.7% of records), 15 pathologies were recorded comprising abscesses, anal fibropapillomatosis, damage of facultative myiasis-producing flies (gasterofilosis), flea, furunculosis (dogs), horseflies, “pedero” or interdigital dermatitis in sheep caused by *Dichelobacter nodosus* (it is a disease, or more precisely an infectious syndrome, of sheep characterized by exudative inflammation followed by ischemia and subsequent tissue necrosis of the hoof, which may result in detachment of the horny case), lice, lice of poultry, mange (soroptic) in pigs, mange or cutaneous acariasis, parasites (indefinite), pimple crusts, small tumors, and tick. Gasterophilosis is a primary myiasis caused by infestation by arthropod larvae of the genus *Gasterophilus*. Adult flies are hairy and similar in size and appearance to honeybees.

Fourteen different pathologies have been registered that affect the digestive system (12.3% of records): anorexia, feeding and nutritional management of dairy cattle, bloat in ruminants (ruminal tympany), pathologies requiring the use of cathartics, colic, detoxification, diarrhea (indefinite), indigestion, enteropathies, hoarseness (it is caused by eating cereal spikes with awns that get stuck in the gums and inner edges of the lips and cheeks producing inflammation, ulceration, and erosion), rumination (alterations), stomatitis, swollen gums, and neonatal diarrhea in ruminants that it alone covers 4% of the records.

Five pathologies or procedures were mentioned by our informants related with the reproductive system (c. 6% of records): abortion (spontaneous or induced by abortifacients), abnormal parturition, events or dystocia, placental retention, and sexual stimulation. Parturition is the expulsion of a fetus (and its membranes) from the uterus through maternal passages by natural forces, and in such a state of development a fetus is capable of independent life. It is more likely to proceed successfully without than with human interference in the great majority of cases. Retained placenta is one of the undesirable sequelae of parturition. Retention of fetal membranes is more common in cows. Normally, membranes should be expelled within half an hour to 4–5 h after a calf is born, but because of the intricate cotyledonary junction in cattle, they are often retained for long periods (94).

For the musculoskeletal system (4.7% of records), eight troubles are reported: bruises (indefinite), disorders of the hoof, fractures, hernias, inflammation, painful legs, tendon contracture, and twisted hoofs. “Escarzo” or “escalzo” is mentioned by interviewed shepherds. “Escarzo” is ordinarily produced by a bad shoe or by treating the hoof badly, or by excessively long journeys; when it manifests, it is necessary to remove the red part and place a horseshoe so that the sensitive part does not receive any pressure (106). Twisted hoofs, a pathology also known as podophlegmatitis, is an inflammation of the keratinous tissue of the foot, with a painful deformation of the hoof.

For generalized conditions (2.65%), the 10 categories recorded were notably infectious diseases: anthrax, canine distemper, contagious agalactia, fever (indefinite) and foot-and-mouth disease, honey bee infections (diseases of bees), infection (indefinite), wolf bite (rabies transmission), and type D Enterotoxemia. Bees represent a very particular type of transhumance, since the seasonality of blooms at different altitudes forces a beekeeper to move hives following patterns parallel to the traditional ones of transhumance cattle. Although bee diseases are not found in many veterinary treatises, some dedicate chapters to these diseases and their treatment (107). Here, we recorded an indefinite category: “honey bee infections” (0.4%).

In the field of eye diseases and disorders, we recorded several pathologies, but the single one pharmacologically treated by the interviewed shepherds is cloudy (eye) (0.3%), which is an infectious keratitis due to Rickettsiae (92, 94).

Management and nutrition: pain relief (0.7%) is the major item recorded from this large group, although the management of new born chickens (administering black pepper to fight low temperature), inducing ewe to adopt the lamb of others and to raise it (0.7%), feeding and nutritional management of dairy cattle (0.7%), and early weaning (0.6%) could also be included here.

Finally, only pharyngitis (3.6%) was mentioned concerning the respiratory system and urinary retention (0.9%) with respect to the urinary system.

However, the equivalence with pharyngitis of the emic term “resfriado” in livestock is extremely ambiguous. In lambs and rams, it is usually oestrosis caused by the parasite *Hypoderma bovis* (Linnaeus, 1758) (=*Oestrus bovis* Linnaeus, 1758). In cattle, the term colds means malignant catarrhal fever or contagious rhinitis. The term distemper can be a synonym of cold among the shepherds. In horses, it can be mumps or equine adenitis caused by *Streptococcus equi* (Sand and Jensen, 1888).

### Medicinal and Toxic Species, Ingredients, and Formulations

Two hundred and two species and substances, of which most are plants, have been recorded from the interviews (Supplementary Table 1). The knowledge that involves a greater number of species is related to toxicity (poisons, insecticides, repellents, and ichthyotoxic) with 130 species (113 of plants, 620 records, nine animals, 24 records, four fungi, and two algae). In the case of veterinary treatments, with 108 species, plants continue to be the most numerous (83 species and 676 records), followed by animals (13 species and 49 records), minerals (7 and 49), and organic chemical substances (2 and 39). There is a group of 18 taxa that, despite being considered toxic, are also used locally in the treatment of livestock diseases, thus appearing in both categories.

Different parts of the plants are the most frequently mentioned ingredients or resources, with plants as a whole being the most cited, followed by aerial parts in bloom or not, and the bark. Materials of mineral or animal origin are used less frequently (Table 1).

**Table 1:** Frequency of plant parts among the recorded remedies.

**Parts**	**Records**	**%**	Species^*^	**%**
Whole plant	384	26.8	92	45.5
Aerial parts blooming	240	16.7	46	22.8
Aerial part	149	10.4	42	20.8
Bark	94	6.6	7	3.5
Leaf	65	4.5	20	9.9
Branch	62	4.3	13	6.4
Oil	40	2.8	1	0.5
Root	30	2.1	10	5.0
Salt^**^	28	2.0	1	0.5
Flower	26	1.8	8	4.0
Vinegar	25	1.7	1	0.5
“Miera” resin	23	1.6	1	0.5
Seed	21	1.5	12	5.9
Bulb	20	1.4	3	1.5
Fruit	19	1.3	11	5.4
Stem	19	1.3	9	4.5
Fleshy cone	19	1.3	5	2.5
Whole animal^**^	16	1.1	6	3.0
Lard	15	1.0	1	0.5
Bud	14	1.0	2	1.0
Poison	13	0.9	5	2.5
Snake shed skin	11	0.8	2	1.0

* *Note that one single plant species can contribute with more than one of the different recognized parts*.

** *Other ingredients of animal or mineral origin*.

These ingredients belong to 92 different families of plants, animals, algae, fungi, and bacteria, and categories of mineral substances and synthetic chemicals. As for families of plants or animals that provide a greater number of records as remedies, Lamiaceae stands out with great difference in terms of records (198, 13.8%), and different species used (29) followed by Fabaceae (118, 8.2%, and 16) (Table 2). Asteraceae follows in terms of number of species (16) but is relegated to 12th place in terms of number of records despite its high number of species in the Spanish flora (68).

**Table 2:** Plant (and other) families more often present in the study.

**Family**	**Main categories**	**Records**	**%**	**Species**	**%**
**Plants**					
Lamiaceae	Veterinary, Parasite control	198	13.8	25	12.4
Thymelaeaceae	Veterinary, Parasite control, Ichthyotoxic	131	9.1	1	0.5
Fabaceae	Veterinary, Toxic	118	8.2	16	7.9
Rutaceae	Veterinary	84	5.9	3	1.5
Cistaceae	Veterinary	75	5.2	4	2.0
Apocynaceae	Veterinary, Parasite control	44	3.1	2	1.0
Oleaceae	Veterinary	44	3.1	2	1.0
Plumbaginaceae	Veterinary, Parasite control	41	2.9	1	0.5
Cupressaceae	Veterinary	40	2.8	3	1.5
Scrophulariaceae	Ichthyotoxic	38	2.7	4	2.0
Apiaceae	Veterinary; Toxic	37	2.6	6	3.0
Asteraceae	Veterinary	36	2.5	16	7.9
Fagaceae	Veterinary	36	2.5	5	2.5
Adoxaceae	Veterinary	33	2.3	3	1.5
Vitaceae	Veterinary	30	2.1	1	0.5
Malvaceae	Veterinary	29	2.0	2	1.0
Solanaceae	Veterinary	29	2.0	7	3.5
Poaceae	Veterinary	25	1.7	5	2.5
Aquifoliaceae	Veterinary	23	1.6	1	0.5
Asparagaceae	Parasite control	20	1.4	2	1.0
Paeoniaceae	Toxic	18	1.3	2	1.0
Anacardiaceae	Veterinary	16	1.1	2	1.0
Cucurbitaceae	Veterinary	16	1.1	2	1.0
Caryophyllaceae	Veterinary	13	0.9	3	1.5
Ranunculaceae	Toxic	12	0.8	3	1.5
Lauraceae	Parasite control	9	0.6	1	0.5
Ericaceae	Veterinary	8	0.6	2	1.0
Pinaceae	Veterinary	7	0.5	3	1.5
Polygonaceae	Veterinary	7	0.5	2	1.0
Rosaceae	Veterinary, Toxic	6	0.4	3	1.5
**Minerals**					
Metal halide	Veterinary	29	2.0	1	0.5
**Animals**					
Suidae	Veterinary	17	1.2	1	0.5
Lamprophiidae	Veterinary	9	0.6	1	0.5
Viperidae	Toxic	9	0.6	1	0.5
Buthidae	Veterinary, Toxic	7	0.5	1	0.5
Colubridae	Toxic	7	0.5	2	1.0
**Organic chemicals**					
Phenolic	Veterinary	9	0.6	1	0.5
Subtotals		1310	91.4	140	69.3
Others		123	8.6	62	30.7
Totals		1433	100	202	100

*Among the 202 species identified, 164 are plant, 19 are animals, seven are minerals, five are fungi, three are organic synthetic chemicals, two are algae, and one is bacteria. Among the 56 families of mainly plants and the other categories above, the only represented here are those with more than five records*.

Rutaceae and Cistaceae present less species than the Asteraceae, Rosaceae, and Apiaceae families, but they almost tripled these in terms of number of records. The overrepresentation of Thymelaeaceae in the interviews (131 records) must be underlined, which is due to the single species of this family that is used, *Daphne gnidium*, and to its popularity as a veterinary remedy, together with its use in the control of parasites and even as ichthyotoxic (Table 2). This made us recall the debate on the differential ethnopharmacological relevance of plant families raised by Moerman (108) and Weckerle et al. (109).

Lamiaceae is not only the most relevant plant family in terms of number of species but also in terms of frequency of use. Among the 36 most commonly used plant species, c. one fourth are Lamiaceae (Table 3). Amid these, *Rosmarinus officinalis* and *Mentha cervina* represent more than 2% of the records.

**Table 3:** Most frequent species in the veterinary of transhumance in Castile La-Mancha.

**Species**	**Main categories**	**Records**	**%**
*Daphne gnidium* L.	Veterinary, Parasite control, Ichthyotoxic	131	9.1
*Cistus ladanifer* L.	Veterinary	70	4.9
*Erophaca baetica* (L.) Boiss.	Toxic	62	4.3
*Ruta montana* (L.) L.	Veterinary	55	3.8
*Rosmarinus officinali*s L.	Veterinary	45	3.1
*Olea europaea* L.	Veterinary	43	3.0
*Plumbago europaea* L.	Parasite control	41	2.9
*Nerium oleander* L.	Veterinary	38	2.7
*Mentha cervina* L.	Parasite control	33	2.3
*Sambucus nigra* L.	Veterinary	31	2.2
*Vitis vinifera* L.	Veterinary	30	2.1
NaCl (Sodium Chloride)	Veterinary	29	2.0
*Malva sylvestris* L.	Veterinary	24	1.7
*Ilex aquifolium* L.	Veterinary	23	1.6
*Mentha pulegium* L.	Parasite control	21	1.5
*Juniperus oxycedrus* var. *badia* H.Gay	Veterinary	20	1.4
*Retama sphaerocarpa* (L.) Boiss.	Veterinary	19	1.3
*Drimia maritima* (L.) Stearn	Parasite control	18	1.3
*Quercus rotundifolia* Lam.	Veterinary	18	1.3
*Ruta angustifolia* Pers.	Veterinary	18	1.3
*Juniperus oxycedrus* L.	Veterinary	17	1.2
*Sus domesticus* Erxleben, 1777	Veterinary	17	1.2
*Bryonia cretica* subsp. *dioica* (Jacq.) Tutin	Veterinary	15	1.0
*Lupinus angustifolius* L.	Toxic	15	1.0
*Paeonia broteri* Boiss. & Reut.	Toxic	15	1.0
*Pistacia terebinthus* L.	Veterinary	14	1.0
*Marrubium vulgare* L.	Parasite control	13	0.9
*Oenanthe crocata* L.	Toxic, Ichthyotoxic	13	0.9
*Verbascum rotundifolium* Ten.	Ichthyotoxic	13	0.9
*Ocimum basilicum* L.	Parasite control	12	0.8
*Dictamnus hispanicus* Webb ex Willk.	Parasite control	11	0.8
*Ocimum minimum* L.	Parasite control	10	0.7
*Sideritis tragoriganum* Lag. subsp. *mugronensis* (Borja) Obón & Rivera	Veterinary	10	0.7
*Stipa tenacissima* L.	Veterinary	10	0.7
*Thymus mastichina* (L.) L. subsp. *Mastichina*	Parasite control	10	0.7
*Verbascum sinuatum* L.	Ichthyotoxic	10	0.7
		974	68.0
Others		459	32.0
Totals		1,433	100

*Among the 202 recorded species, the only represented are the 21 species with more than 10 records*.

Most of the ingredients came from local wild species (79% of taxa), followed by cultivated plants (12.4%) and livestock (c. 3%), and only a few are minerals (3.5%), chemicals (1.5%), or imported species (0.5%). The proportion of imported species is significantly lower than the one recorded in ethnopharmacological studies of the same area (62). Most taxa are Mediterranean (51%), widespread (30%), or European endemics (17%).

It is relevant to notice that the ingredients analyzed here were mainly those employed in conventional formulations administered topically, orally, or by means of inhalations, or used in environmental quality control (disinfection, parasite control) and not those merely used for rituals, even if these had a curative purpose. However, there were some (*Daphne gnidium* stem bark, *Stipa tenacissima* leaves) that were braided and simply carried or tied, with the purpose of preventing or curing different pathologies and traumatisms. The braiding of the strips of “torvisco” (*Daphne gnidium*) entails a certain danger given the toxicity of the plant, and in sensitive people, it can cause irritation (110). This could be related to the activity that is expected from these strings when they are tied to the tail or neck of the animals.

The complexity of the formulations recorded is variable but low, with a maximum of five ingredients. Monospecific preparations prevail (83.2% of records), followed at a long distance by those involving two species (9.4%), three (5.7%), four (1.4%), and finally, five (0.3%) (Table 4). Most of these formulations have only been described in a single interview by an informant single or group, which confirms the vestigial character of the information we have collected and the heterogeneity and adaptive value of the culture from which they come.

**Table 4:** Relevant complex formulations.

**Complex formulation**	**Pathologies treated**	**Records**	**Ingredients**	**Times mentioned**
Dissolve salt in vinegar	Wound healing, bloat in ruminants, cloudy (eye), pharyngitis, stomatitis	24	2	12
Mix rosemary with rockrose (*Cistus ladanifer*) and “chaparro” (*Quercus rotundifolia*) tanning and bring to boil	Disorders of the hoof, wound healing	23	3	8
Poultice of crushed mallow and lard (pork fat)	Disorders of the hoof	21	2	11
Rockrose and rosemary decoction	Hoof care, interdigital dermatitis in sheep caused by *Dichelobacter nodosus*, wolf bite, wound healing	17	2	9
Fry rue in olive oil	Wound healing	17	2	9
Crush broom (*Retama sphaerocarpa*) and mix with vinegar and salt	Bruises (indefinite), disorders of the hoof, hoof care	9	3	3
Use brine with lactobacilli, where they keep the olives	Indigestion, rumination alterations	8	3	3
Fry onion, bay leaf and scorpion in olive oil	Urine retention	8	4	2
Mix juniper galbuli (*Juniperus oxycedru*s) with salt or mulled wine	Placental retention	6	2	3
Fried “cosco” (hoof scrapes) with olive oil	Wound healing	6	2	3
Mix lard with a decoction of rockrose and rosemary	Wound healing	6	3	2
Snake shed skin sandwich with bread	Pain relief, pharyngitis	6	2	3

*Among the 46 recorded formulations with two or more ingredients, the only represented are those with six records or more*.

Direct use (without prior preparation; 55% of records), decoction (14.5%), and grinding (5.1%) are the most frequent processes that the ingredients undergo before being administered.

The routes and forms of administration of the preparations are very varied, more than thirty, with the most frequent being those administered orally (28% of records), followed by the environmental type (26%), in which the product is deposited in the cattle bedding, hung in the enclosures, or burned to produce smoke. Application on or washing of wounds (17%) follows in importance. Application in the form of ointment or poultice is also relatively frequent (9.3%), followed by tying various materials to the tails of lambs to cut diarrhea (3.5%) or massaging the animals' bellies with sticks, known as “magnar” (2.8%).

### Relevant Species With Ethnoveterinary Uses

#### Thymelaeaceae

As a polyvalent species, *Daphne gnidium* L. (131 records, 9.1%) stands out and contributes one 10th of the records of our study. It is included by our informants in the category of plants poisonous to animals and is a source of fish poisons. In the field of control of parasites, it is used for flea and facultative myiasis-producing flies, and as a general repellant. It is a customary practice to tie a cord of “torvisco” to the lamb tail in case of neonatal diarrhea in ruminants. This plant is also used in the treatment of placental retention, fractures, wound healing, canine distemper, fever (indefinite), diarrhea (indefinite), and general enteropathies. Finally, burnt is used to smoke hives in case of honey bee infections (diseases of bees; Supplementary Table 1).

Tying “torvisco” cords to the tails of lambs and goats with diarrhea was also done in Murcia (111). A study conducted in Doñana National Park investigated the relationship between feeding selection by goats over 3 years. Goats showed selective feeding, *Daphne gnidium* was never grazed (112). This plant was used to combat water snakes, which was achieved by putting it in lakes where domestic animals went to drink after transhumance and was also used by fishermen to catch eels from streams (113). At species level, the taxon mentioned with the highest number of references in ethnoveterinary studies is *Daphne gnidium* L. in Spain, orally and topically administered (46).

It is used in different countries, especially in Morocco, to treat several disorders in humans. It is also used as an insecticidal and anti-parasitic (114).

#### Cistaceae

Among the species of fundamentally therapeutic use, *Cistus ladanifer* L. (70 records, 4.9%) stands out by far. The various recorded uses for this plant range from fly control to healing wounds and wolf bites (with the risk of transmitting rabies), and include the process of inducing sheep to adopt lambs from other mothers for breeding, treatment of bites specifically of snakes (viper), fractures, hoof disorders, interdigital dermatitis in sheep by *Dichelobacter nodosus* (Beveridge, 1941) Dewhirst et al., 1990, prickly scabs and urine retention, and feeding and nutritional management of dairy cattle.

Widely used in the Arribes del Duero, for bone fractures of sheep and goat limbs, after resetting the bone, leaves of *Cistus ladanifer* L., glued with its own exudate (labdanum), are placed on the limb, which is splinted using two branches (without leaves) tied with twine, also used to treat wounds of cattle and horses (42). The essential oil of *C. ladanifer* showed remarkable antimicrobial activity (115).

#### Fabaceae

*Retama sphaerocarpa* (L.) Boiss. (19 records, 1.3%): although not extremely dangerous, this species is also included by the shepherds in the category of plants poisonous to animals, goats in particular. It is thrown in the cattle bed to control fleas. Crushed, directly, or in decoction and poultices, it is externally applied to treat mange (soroptic) in pigs, wound healing, bruises, and disorders of the hoof, fractures, tendon contracture, and for curing bites of facultative myasis-producing flies. Orally, it is administered in cases of indigestion and rumination alterations.

#### Rutaceae

*Ruta montana* (L.) L. (55, 3.8%): rue is a multifaceted species in the culture of shepherds in this region. It is among irritant plants and those poisonous to animals. However, it is useful in fly control and as a general repellent specifically of mosquitoes, moths, and mice. It is in the list of fish poisons. The plant is used to prevent bites in general and those of viper in particular. The plant or its decoction was administered to treat placental retention or to induce abortion. Other veterinary uses recorded include wound healing, pain relief, and treatment of neonatal diarrhea in ruminants and prolapse of the oviduct in poultry.

Its main use is in the gynecological field, but it is also described for treatment of pain, fever, nausea, inflammation, infections, and nervous disorders, among others. These plants have been used for fertility regulation, as an antifertility agent, to control menstrual flow and bleeding, as an abortifacient, and as a contraceptive. These plants have also been tested for use in non-therapeutic approaches, as bio-pesticides in the control of different insect pests proving to be able to reduce infestation (116). In northern Spain (Palencia), frying in oil of *R. montana* is performed to treat mastitis in cattle (117).

*Ruta angustifolia* Pers. (18 records, 1.3%): similar to the above species, it is placed by our informants, in this case from eastern Castilla-La Mancha, among plants poisonous to animals. However, its properties are recognized in the control of fleas, and it is externally applied for wound healing and orally administered, in decoctions, as cathartics, to treat bloat in ruminants, indigestion, and rumination alterations. It is also administered in cases of abnormal parturition events and placental retention.

In El Caurel (Galicia, Spain), Blanco et al. (118) reported similar uses for *Ruta chalepensis* L.

#### Lamiaceae

*Rosmarinus officinalis* L. (45 records, 3.1%): although rosemary is used as a disinfectant of rooms and a general repellant, it is mostly used in ethnoveterinary, especially along the western part of transhumance routes, for wound healing or in hoof care and treatment of fractures, wolf bite, interdigital dermatitis in sheep caused by *Dichelobacter nodosus*, bloat in ruminants, and urine retention. It is relevant in feeding and nutritional management of dairy cattle. When burnt, its smoke is used for honey bee infections (diseases of bees).

In northern Morocco, the powder of rosemary is administered by massaging to goats to treat endo- and ectoparasites. It is widely used for internal parasites (119). In northern Spain (Palencia), they prepare an extract in wine that is given to drink to cattle to treat blood in the urine (117). The aerial part is used in Spain for diarrhea, indigestion, intestinal colic, bloat in ruminants, abdominal pain, liver affections, and as a purgative for equids, pigs, sheep, cattle, and poultry. It is used for urinary retention, and to treat mastitis and other diseases, to facilitate labor, retained placenta, sexual stimulant for livestock, pneumonia (cattle), bruises and contusions, bone fractures, dislocations (equines, sheep, and dog), wounds, and dandruff (livestock in general) (46).

*Mentha cervina* L. (33 records, 2.3%): the blooming aerial part of the plant is noteworthy as a general repellant and particularly in horsefly, mosquito, and fly control. A decoction of *Mentha cervina* is orally administered in cases of bloat in ruminants.

Rodrigues et al. (120) characterized essential oils (EOs) from *M. cervina* and tested their antimicrobial activity against 23 bacterial strains (including multidrug-resistant strains). The EOs were dominated by the monoterpenes pulegone (52–75%), isomenthone (8–24%), limonene (4–6%), and menthone (1–2%). The most effective antibacterial activity was expressed by the EOs against Gram-negative bacteria, *Escherichia coli*, and *Acinetobacter baumanni*. Complex mixtures of EOs were more active than individual aromatic compounds. These results show the potential role of *M. cervina* EOs as antibacterial agents and validate the traditional use of this plant.

Gonçalves et al. (121) evaluated the antifungal activity of Mentha essential oils against *Candida, Aspergillus*, and dermatophyte strains. The antifungal activity of the sample containing lower amounts of pulegone was the highest for dermatophytes, in particular for *Epidermophyton floccosum* (Harz; Langeron and Miloch). *Mentha cervina* oils with low pulegone content may be an alternative as antifungal agents in dermatophytosis.

El Zayyat et al. (122) tested on adults and larvae of *Musca domestica* Linnaeus, 1758, the bioactivity of *M. cervina, Ocimum basilicum*, and *Coriandrum sativum*. *M. cervina* was less toxic and, thus, less effective than basil or coriander.

*Mentha pulegium* L. (21, 1.5%): the blooming aerial part of the plant is remarkable as a general repellant and especially in flea, mosquito, and fly control. A decoction of *Mentha pulegium* is orally administered in cases of bloat in ruminants. Uses of this plant are similar to those of *M. cervina*, as above, and likely due to the active principles both species share, notably pulegone.

In Granada (Spain), this species is widely used as a preventive for postpartum infections and is hung in corrals to repel biting insects (16). In Sardinia, the fresh plant has been used to repel worms from cereal grains and beans (123). In some areas of Catalonia (Spain), a tisane is prepared from this plant together with rice and is used as an antidiarrheal in young calves (37). In equids of some areas of Portugal, it is used for gastrointestinal disorders (124).

In supplementation, trials conducted with pennyroyal (*M. pulegium*) positively affected broiler growth traits and blood parameters, and reduced harmful intestinal bacteria (125).

#### Plumbaginaceae

*Plumbago europaea* L. (41 records, 2.9%): it is renowned among plants poisonous to animals and fish poisons. It is relevant as a general repellant, disinfectant of rooms, and in the control of flies, louse, fleas, and mosquitoes. Occasionally, it is used in foot-and-mouth disease and wound-healing.

Al-Qura'n (126) cited the whole plant as toxic in Jordan and that if eaten it causes abortion and sterility. In Grenada, it is used to combat parasites (16). Occasionally is used in foot-and-mouth disease and wound healing, and as a cicatrizing in Sardinia (127).

Ali-Shtayeh and Abu Ghdeib (128) tested the antifungal activity of extracts of this species and it was effective against dermatophytes. The antifungal activity of plumbagin of this species was studied by Jaradat et al. (129), concluding that *P. europaea* roots are the best source of plumbagin and that the plant extract could represent a potential drug candidate for treatment of dermatophytosis infections in humans and for veterinary use.

#### Cupressaceae

**Figure 4:**
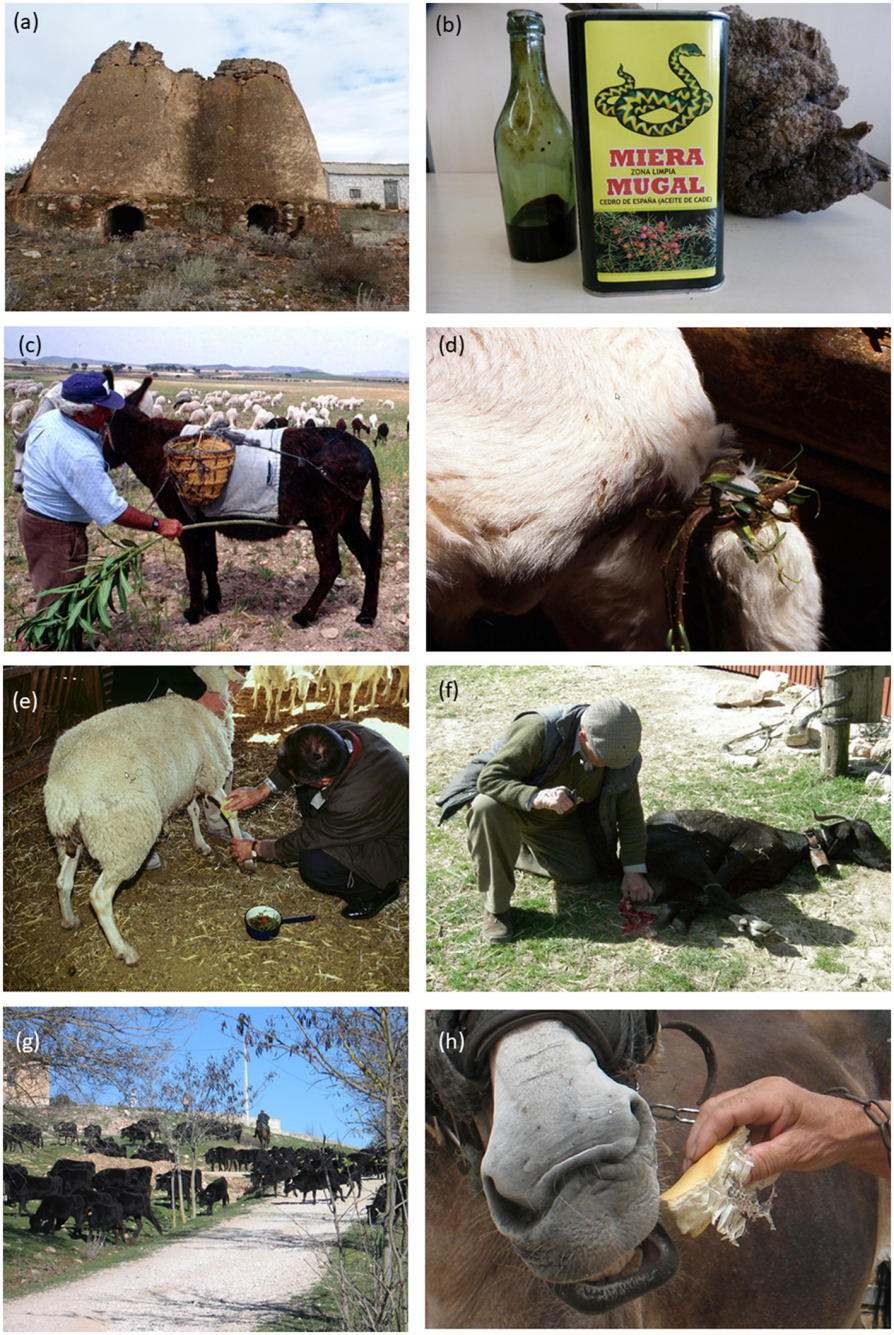
Selected practices and ethnoveterinary ingredients. **(a)** “Miereradel Charco” proto-industrial facility. **(b)** Commercial “miera” or juniper distilled resin. **(c)** Use of an oleander branch to massage a donkey's belly. **(d)** Braid of torvisco branch bark tied to the tail of a lamb to control diarrhea. **(e)** Curing a sheep's foot with a decoction of medicinal plants. **(f)** Treatment of mastitis in the udder of a goat. **(g)** Transhumant cattle crossing Olmeda del Rey (Cuenca). **(h)** Feeding a mule with bread and snakeskin sandwich to treat a cold, Abengibre (Albacete). Photos: J. Fajardo and A. Verde.

*Juniperus oxycedrus* L. (including var. *badia* H.Gay; 39 records, 2.8%): “Miera” or juniper tar is the resin obtained through dry distillation of wood chops from this tree, it was a fundamental remedy for shepherds in the treatment, externally applied, of mange (soroptic) in pigs, mange or cutaneous acariasis, wound-healing, and bites of facultative myiasis-producing flies. Orally administered, the galbuli were used to treat placental retention. Figure 4 displays images of the distillation facilities known as “miereras” and of the commercial “miera,” which is still sold as a snake repellant and is, thus, used in corrals (non-sense because snakes come in to hunt rodents, but the shepherds believed that they steal milk from ewes and goats).

In Spain, cade oil, or galbuli, has been administered orally to treat indigestion, constipation, and liver infections in cattle. In case of conception, pregnancy, and delivery problems, and retained placenta, cade or galbuli oil is administered orally to sheep and goats. For pneumonia and metabolic diseases, internal use of cade oil for livestock is documented (46). In Alentejo, Portugal, external use has been recorded to treat ectoparasites and trauma in equids (124).

#### Adoxaceae

*Sambucus nigra* L. (31 records, 2.2%): bloat in ruminants is treated giving massage at the belly level using a branch of this shrub. Decoctions, mostly of flowers, single or in mixtures are used to treat wounds, bruises, mange or cutaneous acariasis, inflammation, infection and pharyngitis, and failure to thrive in pediatric pet birds.

In Navarra, inflorescence is performed for cow and mule diseases, especially bronchial, smoke inhalations, and the bark in poultice for mastitis (34).

In the Upper Ter River Valley (Catalonia, Spain), it is used in fumigations for mastitis, and the tisane of flowers is used orally for intestinal discomfort and as an antiseptic or, with *Achillea ptarmica* L., as an antidiarrheal (36).

Viegi and Ghedira (130) cited in Italy the use of the water macerate of the bark for gastrointestinal ailments in chickens and ointment to cure cattle; of fumigations of the decoction of the aerial part to reduce inflammation of udders after calving in cattle; of the flower for colds in fumigations; of blooming branches to catch flies, soaked in buttermilk and hung in stables; and of dried flowers heated in olive oil for sore or inflamed body parts in goats and cows.

#### Malvaceae

*Malva sylvestris* L. (24 records, 1.7%): leaves and flowers of this species in this area are directly, or after being crushed or decoction, applied to treat bruises, inflammation and disorders of the hoof, and are administered for bloat in ruminants and pharyngitis.

*M. sylvestris* is one of the most used plants in different areas of Spain for different diseases of livestock such as digestive disorders, respiratory problems, and dermatological conditions (34, 37, 42), or in areas of Italy such as Sardinia (123). It is used as a laxative in horses and cattle, to treat malignant guts, meteorism in cattle (tympanism), to reactivate rumination as a carminative (swelling of the animal), and pig diseases (diarrhea); in smoke inhalations, it is used for catarrh and in poultices for wounds in cattle and horses, and, finally, in infusion for bruises and hematomas (anti-inflammatory) in oxen and horses (34, 37, 42). Gasparetto et al. (131) made a review of the properties of this plant and indicated multiple veterinary uses, phytochemical constituents, and pharmacological activity.

#### Aquifoliaceae

*Ilex aquifolium* L. (23 records, 1.6%): massage with a previously warmed nude branch of this tree is performed to treat bloat in ruminants, enteropathies, and indigestion in a similar way as it is done with oleander branches.

*I. aquifolium* is used in ethnoveterinary dermatology. The plant, hung over cattle, is used to cure scabies in animals, to combat ringworm in cattle, and to cure other skin problems in cows (34). In Switzerland, to prevent or treat ringworm in cattle, twigs of *Berberis vulgaris* L, *I. aquifolium* L., or *Prunus spinosa* L. are hung in a stable (132).

#### Asparagaceae

*Drimia maritima* (L.) Stearn (18 records, 1.3%): the bulb is one of the local fish poisons. Directly or cut in pieces, it is thrown in stalls to control flea and lice or as a general repellant. A decoction is used to clean stalls as a disinfectant and to treat mange (soroptic) in pigs.

#### Fagaceae

*Quercus rotundifolia* Lam. (18 records, 1.3%): decoction of a single plant or in mixture with rockrose is used for wound healing.

#### Cucurbitaceae

*Bryonia cretica* subsp. *dioica* (Jacq.) Tutin (15 records, 1.0%): decoctions of the root are orally administered for stomatitis, anorexia, bloat in ruminants, colic and pharyngitis, and externally applied for wound healing.

#### Anacardiaceae

*Pistacia terebinthus* L. (14 records, 1.0%): its branches are thrown in stables to control parasites and as a general repellent and administered to ewe in case of weaning lactation.

#### Metal Halides

NaCl (sodium chloride) (29 records, 2%): common salt is a usual ingredient in formulations employed by shepherds in Castilla La Mancha to induce adoption by ewe of the offspring of others to raise them, and to treat bruises, inflammation, disorders of the hoof, cloudy eye, contagious agalactia, wound healing, mange or cutaneous acariasis, indigestion, stomatitis, bloat in ruminants, rumination alterations, hoarseness, and pharyngitis.

It should be remembered that there are several inland salt mines along some livestock trails, so the supply of salt could eventually be local (133, 134).

Salt has been added for many ethnoveterinary remedies in the Pyrenees (27): a paste made with *Bryonia* roots and salt was given to equids for indigestion. *Juniperus phoenicea* ashes were added to salt and given as a mineral supplement to animals. Salt was poured into the affected eye of animals with conjunctivitis and in eyes with uveitis in order to promote lacrimation and, thus, clean the eye of pathogens. Wounds were washed with water and salt. Excessive consumption of salt can cause abortion in pregnant ewes. Every 12 days, salt was added to ports to avoid mineral deficiencies in livestock. When goiter occurs in sheep, iodized salt is given.

In the valley of Carranza, when a calf was just born, in order for it to start breathing, it was given a little amount of salt, then the rest of the salt was spread over the surface of its body and it was dragged to its mother. A calving cow was rubbed with vinegar, salt, and egg to warm it up; first, vinegar was poured all over the back, especially at the level of the kidneys, and rubbed with the hand, then salt and some more vinegar were added and continued rubbing vigorously to “warm it up;” finally, an egg was crushed in the back and carefully spread all over it. It was also given water with salt to expel pariahs. For beards (filiform prolongations that come out in the tongue and internal surface of the lips) of bovine cattle, they were cut and cured with a mixture of vinegar and salt, or of wine and salt, throughout several days (28).

Salt is a remedy widely used alone or in mixtures with other ingredients in Sardinia; in cases of foot and mouth disease, vinegar and salt are used for mouthwashes (123), and in Catalonia, salt is used as a disinfectant and to prevent or cure helminthiasis (37).

In Albania, plant-charcoal powder, mixed with salt, is given to ruminants for diarrhea (135).

### Solvents and Base Media

#### Water

Fresh water is the most common extraction and processing medium. It is used in decoctions in which a single species (221 records) or several (66 records) are involved, and infusions (30 records), macerates (17 records), and solutions (2 records). In second place is vinegar (53 records; *Vitis vinifera* L., Vitaceae), followed by olive oil (51 records; *Olea europaea* L., Oleaceae).

In addition to being the most widely used means of extraction, the water from some springs, because of its temperature and mineral content, has been used in the treatment of diseases. Mineral-medicinal waters that abound in Castilla-La Mancha have occasionally been used to wash and treat sick animals as well as people (61, 136, 137). Water from springs and streams was used alone to cleanse sores and wounds, for example, one of us (J.Fajardo) asked his father, linked to the eastern transhumance routes, what they did with the wounds in the mouths of the lambs and sheep, he calls them sores, and they were produced by eating very dry and rough grass. They washed them with clean water and fed the lambs and sheep with softer forage such as alfalfa.

#### Vinegar

Wine vinegar is frequently used as an ingredient in formulations, often with salt, although in some cases it is used alone. In Castilla-La Mancha, it is used externally to treat bruises, disorders of the hoof, cloudy eye, wound healing, mange or cutaneous acariasis, and orally administered in cases of placental retention, anthrax, contagious agalactia, indigestion, stomatitis, and bloat in ruminants, hoarseness and pharyngitis.

In Sardinia, Piluzza et al. (123) recorded several ethnoveterinary uses of vinegar: two cloves of garlic, crushed, in half a liter of vinegar are offered as food to treat intestinal worms in sheep; udder massage with vinegar for mastitis; insecticide mixing vinegar and olive oil applied and massaged on the skin of sheep; vinegar or vinegar with salt for mouthwashes in sheep.

In Campoo (Cantabria, Spain), vinegar is used to treat renal colic in cows; after bathing the animals in soap and vinegar (to warm them up), the kidney area was covered with a sheepskin. In the Basque Country, after cleaning the legs of a sick cow with a mixture of vinegar and salt, lard was applied as an insulating product (47).

Vinegar is widely used in some areas of the Pyrenees; in the Aragonese area, it is used to treat myiasis by applying vinegar to the affected area. For mastitis, it is used directly or mixed with “buro” (sandy clay). For foot and mouth disease, honey with vinegar was used around a stick as a lollipop to give to livestock (27). In some Catalan valleys, it is used as anticatarrhal, diuretic, antiseptic, and tranquilizer (36).

#### Olive Oil

Olive oil is a major remedy for different pathologies according to shepherds of Castilla-La Mancha and is a base for numerous medicinal preparations. It is used, orally administered, to treat anthrax, bloat in ruminants, indigestion, rumination alterations, and urine retention, and it is externally applied to treat damages from facultative myiasis-producing flies, inflammation, and mange or cutaneous acariasis, and for wound-healing, tick control, and pain relief.

#### Others

Lard (36 records; *Sus domesticus* Erxleben 1777, Suidae) is a base for healing ointments, with other ingredients, used to treat abscesses, disorders of the hoof, furunculosis in dogs, mange or cutaneous acariasis, and wound-healing.

Olive brine, with corresponding lactobacilli (12 records), is another solution administered orally to treat indigestion and bloat in ruminants.

Distilled alcohol is used, either that sold in pharmacies with a purity of 96%, or high-proof liquors such as brandies, indistinctly, but occasionally (3 records).

### Parasite Prevention and Control

One of the fields most widely covered by the responses collected in our study (315 records) is that of prevention, protection, and control of parasites, flies, mosquitoes, and moths.

The procedures are quite simple, since they consist of hanging viscous plants in prominent places in an area where cattle are housed or sleeping and in which various insects stick, or, also, hang very aromatic species containing repellent substances or insecticides. Another possibility is to throw these plants on the ground or in the bedding of cattle or even cook them and throw water in the same place.

At the level of prophylaxis, it is worth mentioning the bedding of corrals, it is not the case of transhumance livestock, but it has been considered very important by shepherds that cattle have good bedding, with dry straw, to prevent many diseases. This bedding substratum is the appropriate place to throw different repellent plant species and other substances.

Control of fleas (Siphonaptera Latreille, 1825), red mites [*Dermanyssus gallinae* (De Geer, 1778)] and lice (Phthiraptera Haeckel, 1896), as well as other hematophagous parasites, is carried out fundamentally with the use of *Ballota hirsuta* Benth., CaO, *Daphne gnidium* L., *Drimia maritima* (L.) Stearn, *Eryngium campestre* L., *Juglans regia* L., *Lavandula pedunculata* (Mill.) Cav., *L. stoechas* L., *Marrubium vulgare* L., *Mentha aquatica* L., *M. pulegium* L., *M. spicata* L., *M. suaveolens* Ehrh., *Picnomon acarna* (L.) Cass., *Plumbago europaea* L., *Retama sphaerocarpa* (L.) Boiss., *Ruta angustifolia* Pers., and ashes of *Salsola kali* L. directly or after maceration or decoction; in these cases, processing water is also used, as well as hanging hoopoe carcasses (*Upupa epops* Linnaeus, 1758; Supplementary Table 1).

The use of synthetic organic compounds was relatively frequent depending on the context, including 2-(4-Bromophenyl) naphthalene and Zotal^®^. Although we have not recorded this use in the interviews, the “miera,” distilled tar of *Juniperus oxycedrus*, was and is hitherto used in Albacete and Cuenca as a snake repellent in corrals, and it is put on doors and windows. It is yet to be marketed for this purpose (Figure 4).

Moth (Heterocera) control is also based on a repertoire of plants, among which several are in the previous category (*Lavandula latifolia* Medik., ^*L*^*. stoechas* L.,) but others (*Crocus sativus* L., *Dictamnus hispanicus* Webb ex Willk., *Juniperus thurifera* L., *Laurus nobilis* L., *Nicotiana rustica* L., *N. tabacum* L., *Pinus nigra* Arnold subsp. *salzmannii* (Dunal) Franco, *P. pinea* L., *Ruta montana* (L.) L., and *Thymus vulgaris* L.) are exclusive (Supplementary Table 1).

They also use numerous species as repellents, generally for insects, and specifically for flies and mosquitoes. The following are worth noting: *Daphne gnidium* L., *Drimia maritima* (L.) Stearn, *Eryngium bourgatii* Gouan, *Jasminum polyanthum* Franch., *Juglans regia* L., *Lavandula latifolia* Medik., *Lupinus albus* L., *Medicago sativa* L., *Mentha* × *rotundifolia* (L.) Huds., *M. aquatica* L. var. *citrata* (Ehrh.) Fresen., *Mentha cervina* L. (25 records), *Mentha pulegium* L. (17 records), *Mentha suaveolens* Ehrh. (six records), *Nerium oleander* L., *Ocimum basilicum* L. (13 records), *Ocimum minimum* L. (11 records), *Pistacia terebinthus* L., *Plumbago europaea* L., *Quercus faginea* Lam. subsp. *faginea, Quercus faginea* subsp. *broteroi* (Cout.) A.Camus, *Rosmarinus officinalis* L., *Ruta montana* (L.)L., *Salsola vermiculata* L., and *Thymus mastichina* (L.) L. subsp. *mastichina*. The use of the aforementioned species as repellents is also based on the effect produced on the environment by the release of volatile substances from plants that are hung or thrown on the floor of a fold.

Gadflies or horseflies (tabanidae): the horsefly family is large and important, as females are bloodsuckers, and some inflict a very painful bite on humans. Several genera and species can mechanically transmit diseases to livestock and humans (94). The gadfly repellent recorded is *Mentha cervina* L.

To eliminate mosquitoes from human and livestock habitations, “belesa” plants, notably *Peganum harmala* L. and *Plumbago europaea* L., were burned in cans. The toxic smoke eliminated insects from a room, which could be inhabited after a few minutes.

Brooms were made with the aerial parts of *Chondrilla juncea* L. to remove parasites from livestock by brushing and especially mosquitoes.

Various plants were hung to be used as glutinous fly traps: *Arbutus unedo* L. (shoots), *Asparagus acutifolius* L., *Cistus ladanifer* L., *Silene muscipula* L. and *Tamarix gallica* L.

Aqueous extracts of *Daphne gnidium* were proved to be toxic to insects. This led Benhissen et al. (138) to include *Daphne gnidium* among plants with important insecticidal effects on biological mosquito control. Preliminary toxicity tests on *Culex pipiens* Linnaeus, 1758, have confirmed its toxicity to these vectors, and with lower doses of the aqueous extract of *Daphne gnidium*, a reduction in fertility and fecundity of treated adult female larvae was observed.

### Traditional Knowledge of Poisonous Plants and Animals

#### Toxic Plants

In the context of animal health, knowledge of various toxic and poisonous species is a prerequisite for safe grazing. There are catalogs and manuals of toxic flora, specialized such as those of San Andrés et al. (139) and González-Stuart (93), or guides of useful and toxic plants for the Iberian Peninsula (140). Types of animal reactions to toxic plants include dermatitis (contact irritation) and tissue inflammation (from irritating chemical compounds released by a plant). Irritant poisons produce acute abdominal pain, vomiting (when possible), purging, rapidly developing general collapse, and often unconsciousness, sometimes preceded by convulsions (93, 94).

Herders often recognize as toxic or poisonous not only species that contain toxic chemicals (141) but also those that can act as a reservoir of pathogens or mark the existence of dangerous places such as the “lobadea” grass for the “cursed fields” of anthrax and the “basquilla” grass linked to enterotoxemia caused by *Clostridium perfringens*.

Among toxic plant species, the most cited in the interviews should be highlighted: *Erophaca baetica* (L.) Boiss., *Lupinus angustifoliu*s L. (15), and *Oenanthe crocata* L. (13).

*Erophaca baetica* (L.) Boiss (=*Astragalus lusitanicus* Lam.; 62 records, 4.3%) is the toxic species most frequently cited by the shepherds interviewed as toxic for sheep, goats, and cattle, and, in general, to animals and humans. Numerous articles have been published dealing with the toxicity of this plant to livestock, especially sheep and goats (142–146). The responsible compound is not clear. Hypophorin, an α-N,N,N-trimethyltryptophan betaine, was isolated for the first time from this species. This alkaloid was characterized by NMR and MS analysis. Although hypophorin was reported to be a convulsant poison, to verify its toxicity, Bel-Kassaoui et al. (147) synthesized the compound and tested it in goats, showing that hypophorin is not toxic to goats even at a high dose of 2 g/kg by oral administration.

*Lupinus angustifoliu*s L. Lupinism or lupine alkaloid intoxication is an acute intoxication characterized by central nervous symptoms. It is caused by consumption of large quantities of lupines, with a high alkaloid content (0.7–1.8%), in a short period of time. It is not frequent, because animals reject them because of their bitter taste (148).

*Oenanthe croccata* L.: its roots contain the long-chain acetylenic alcohols oenanthotoxin, oenanthenol, and oenanthenone of great convulsant power. The lethal dose for small ruminants is estimated at 2 g of fresh tubers /kg body weight (148).

*Paeonia broteri* Boiss. and Reut. (15 records, 1%) is especially cited for the irritating nature of its flowers. It is known among plants poisonous to animals, and an irritant. However, it is administered in case of placental retention.

*Nerium oleander* L. (38 records, 2.7%): it is included by our informants among plants poisonous to animals, and has irritant flowers. They report its bitter taste, and that livestock do not graze on it. However, it is used as a general repellant, and it is mixed with salt and vinegar to wash affected parts in cases of mange or cutaneous acariasis. Shoots were occasionally tied to a lamb's tail to treat neonatal diarrhea in ruminants. Massage with branches of this shrub was practiced as a pain reliever in cases of enteropathies, indigestion, and bloat in ruminants, horses, mules, and donkeys.

Curiously, in search engines, when associating this species with veterinary medicine, articles on its toxicity appear primarily; however, associated with ethnobotany, numerous studies are found in which uses of this plant are recorded in addition to its toxicity.

Oleander poisoning can be fatal to humans, animals, and even some insects (149). The high toxicity of oleander to livestock and its transfer to milk and dairy products have been reported, suggesting a potential risk to consumers (150). In Europe, horses are particularly at risk of poisoning by, among other species, Nerium oleander (oleander) (151).

Oleanders contain cardenolides in their tissues that are able to exert positive inotropic effects on the hearts of animals and humans, and their leaves contain mainly oleandrin. Severe depression, anorexia, ruminal atony, diarrhea, serous nasal discharge, tachycardia, and irregular heartbeat were the most common clinical signs of oleander poisoning in cattle.

In Italy, oleander branches are stuck in the ground to poison moles, and in Sicily, the flowers are scattered on the ground in cockroach-infested areas (113). Also, in Granada (Spain), oleander stems were used in a healing ritual for digestive disorders, in which crosses were made on the animal's belly while reciting a healing prayer (16).

#### Plants as Reservoirs of Pathogens

*Lupinus angustifolius* L. (15 records, 1.0%) is reported by our informants to be toxic to *Bos taurus* Linnaeus, 1758 (cow), *Capra hircus* Linnaeus, 1758, *Oryctolagus cuniculus* (Linnaeus, 1758), *Ovis aries* Linnaeus, 1758, and, in general, to animals and humans. It is one of the plants reported in the area to cause “basquilla” or type D enterotoxemia. Among the species attributed by our informants to be causing “basquilla” (type D enterotoxemia) when ingested by cattle are *Bituminaria bituminosa* (L.) C.H.Stirt., *Delphinium staphisagria* L., *Lupinus angustifolius* L., *Potentilla erecta* (L.) Raeusch.,*Sanguisorba verrucosa* (Link ex G. Don) Ces., and *Vincetoxicum hirundinaria* Medik. Although several of these species contain toxic substances ranging from alkaloids to lupinanes, it is very likely that in all cases, the triggering factor is the presence of toxins from *Clostridium perfringens* (Veillon and Zuber, 1898) Hauduroy et al., 1937 spores in the plant or in its surroundings' soil.

Even more remarkable is the relationship between “lobadeas” grasses and “lobado” or anthrax caused by *Bacillus anthracis* Cohn 1872. “Lobado” is a zoonosis that caused numerous problems in shepherds, often becoming fatal. When it affected people, it was called anthrax, and in animals, it was also called “spleen.”

A“Lobadea” plant grows mainly in peat bogs and can be several species, although other species from ravines, streams, and the Guadiana River itself are also included, such as *Flueggea tinctoria* (L.) G.L. Webster, *Lupinus angustifolius, Ononis spinosa* L., and *Potentilla erecta* (Supplementary Table 1). More than the nature of the grass, it is the places, which unfortunately have traditionally been used to dump waste, that pollute, possibly because at one point carcasses of animals that died of anthrax were deposited in them, creating a reservoir of spores.

According to our informants, their goats died when they ate “hierba lobadea” during the second regrowth and not the first time the grass was grazed. They ate it, then they sprouted again, and when they ate it again, it is when it became toxic. To interpret this observation, we must consider that *Bacillus anthracis* itself is invasive fundamentally when inhaled. An animal, when grazing on contaminated soil covered with dry dust, inhales the spores (152). Hence, the first time an infected area is grazed, with the grass completely covering the ground, which is wet, its moisture significantly reduces the chances of infecting the livestock; thus, it is perceived as safe. However, later, with the soil devoided of grass coverage and exposed for days to sun and wind, drought and dust can arise, and with them are facilities for spore dispersal.

#### Poisonous Animals

Our informants have pointed out eight different species of animals as poisonous (Supplementary Table 1). Some, such as vipers and scorpions, are undoubtedly dangerous to livestock and humans, while others, such as hedgehogs and salamanders, present peculiarities that merit comment.

Among species of poisonous animals recognized as dangerous by herders are various scorpions, and *Buthus ibericus* (Lourenço and Vachon, 2004) [= *Buthus halius* C. L. Koch, 1839], alacrán (Albacete, Viso del Marqués), arranclán (Navalpino), are the commonest in this area. Notwithstanding its toxicity, fried in olive oil, alone or with onion and bay leaves, a scorpion is used to treat urinary troubles in livestock (Supplementary Table 1). This preparation was recorded in other areas by González and Vallejo (153). It is part of the zootherapeutic repertory recorded by Quave et al. (154).

*Vipera latastei* Bosca, 1878, víbora (Albacete, Alcoba, Casas de Lázaro, Cotillas, Lezuza, Riópar, Villaverde de Guadalimar, and Viso del Marqués): *V. latastei* is poisonous and it is found throughout Spain except in the northern fringe (155).

*Salamandra salamandra* (Linnaeus, 1758), tiro (Albacete, Riópar, Villaverde de Guadalimar): its black and yellow design gives it an aposematic coloration that alerts potential predators to its toxicity and bad taste (157). Salamanders' toxic compounds include steroidal alkaloids and tetrodotoxins (158).

*Malpolon monspessulanus* (Hermann, 1804), serpiente (Navalpino): *Malpolon* is a venomous snake. If disturbed, it flees but if it feels threatened; it is more aggressive than others, and before biting, it often hisses in a very characteristic way and raises its head as cobras do (155).

*Erinaceus europaeus* Linnaeus, 1758: in itself, hedgehog is not poisonous, and even several parts of this animal were used for medicinal purposes in Europe (154). However, hedgehog is susceptible and a potential carrier of foot-and-mouth disease (159, 160), and likely infected animals' urine or excrements could be harmful.

*Blanus cinereus* Vandelli, 1797 cucuveo (Tobarra) feeds mainly on insects found on fallen leaves, grounds, or under rocks. It can provide small bites when handled, which can lead people think it is aggressive and poisonous (161, 162).

*Chalcides bedriagai* (Bosca, 1880), eslabón (Carrión de Calatrava): the “eslizón” is a not venomous reptile feeding on arthropods (163). The skink is not poisonous, although a popular belief says so, not only in Castilla-La Mancha. For example, in the northwest of Murcia (Spain), it is believed that it spits, and that its saliva is poisonous, which is not true.

*Hemorrhois hippocrepis* (Linnaeus, 1758), alicántaros (Daimiel), alicántara (Viso del Marqués): it should be noted that *H. hippocrepis* lacks fangs and is not venomous. (155, 156). The alicántara is probably confused with *Vipera latastei*, which is poisonous.

#### Uses of Ichthyotoxic Plants

Fishing with the help of poisonous plants was once common in Castilla La Mancha. Today, this easy and simple method of fishing is prohibited and almost no longer practiced in these areas. Poisonous ingredients, usually leaves, are crushed and thrown into a pond or dammed sections of small rivers and wetlands.

Species used in fishing have toxicity due to saponins and other water-soluble substances that are released into the water by throwing parts of plants containing toxic substances after having crushed them. In the area investigated, the most commonly used sources of ichthyotoxic substances are “torvisco” (*Daphne gnidium* L.), “belesa” (*Plumbago europaea* L.), and different species of mullein (*Verbascum pulverulentum* Vill., *V. rotundifolium* Ten., *V. sinuatum* L., *V. thapsus* L., and *V. virgatum* Stokes). Occasionally, other ichthyotoxic species were mentioned whose toxicity to livestock was also recorded: *Drimia maritima* (L.) Stearn (bulb), *Oenanthe crocata* L. (root), *Ruta montan*a (L.)L. (whole plant), and *Thapsia villosa* L. (root) (Supplementary Table 1).

In Sardinia, crushed roots of *Daphne gnidium* L. were used in fishing and were considered superior to those of other species (164).

Mullein species (*Verbascum*) were often used in the area for illegal fishing. However, they are not markedly toxic to livestock, although the stellate, branched hairs, which cover the leaves so densely, act as a protective coating, providing defense. They produce intense irritation to the mucous membrane of grazing animals that attempt to eat them. The main toxicants affecting the circulatory, respiratory, and nervous systems of fish are saponins, rotenone, and glucosides. Mullein, when thrown crushed into lentic waters, causes fish to have breathing difficulties (165). Among the most important *Verbascum* species that poison fish are *V. phlomoides* L., *V. sinuatum*, and *V. thapsus*. The plant parts generally used are capsules and seeds, which are ground between stones and thrown into water (164). Our informants mention the use specifically of seeds or, alternatively, of leaves and the whole plant (Supplementary Table 1).

The root of *Oenanthe crocata* L. was used in fishing in Sardinia and Portugal (164).

### Relationships Between Main Transhumance Ways and Zones

The main livestock routes (cañadas reales) cross Castilla-La Mancha from the northeast to the southwest, generally in parallel, with a distance of about 400 km between the westernmost and the easternmost, and the longest exceeds 400 km. We included in our study 33 of these with different levels of relevance and information recorded.

Among the studied “Vias Pecuarias” worth of mention for the number of records delivered are Cañada Real Segoviana, Cañada Real de Andalucía (de los Serranos), Cañada Real Soriana Oriental, Cañada Real Galiana, and Cañada Real de la Plata (Figure 1), being all of the above confined to the central and western parts of Castilla-La Mancha, with exception made for Cañada Real de Andalucía (de los Serranos).

The results of the hierarchical classification by means of Ward's minimum variance are disappointing to a certain degree, since the tree we obtained is present together the “cañadas” from which we have a greater number of records regardless of their geographical proximity or other factors. This is mainly due to the non-Euclidean nature of the distances calculated.

**Figure 5:**
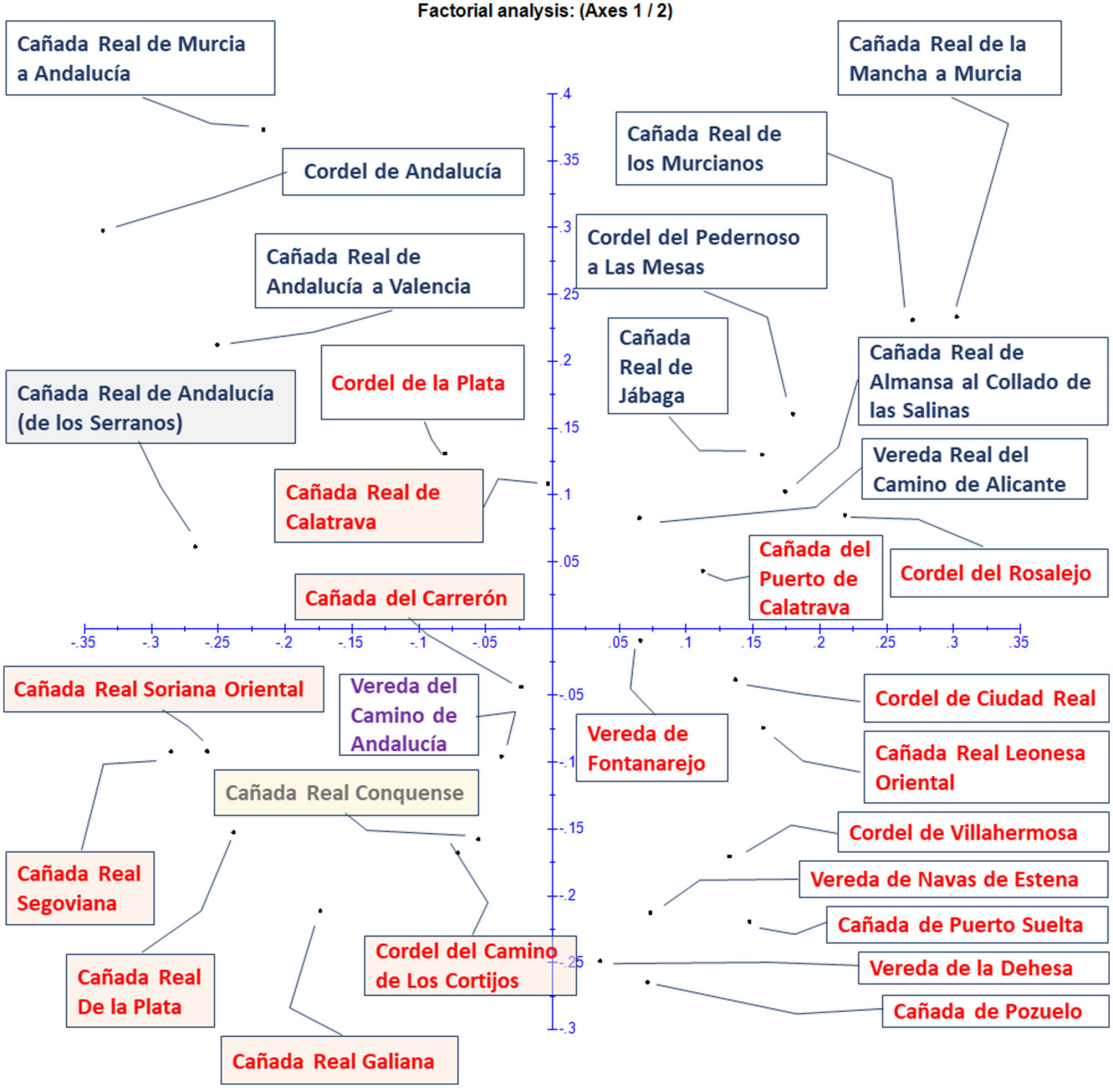
Factorial analysis (PCoA) of relationships between transhumance roads based on the ingredients. Western roads in red characters. Eastern roads in blue characters.

On the contrary, Principal Coordinate Analysis (PCoA), working with the same dissimilarity matrix, provides a graphic representation full of meaning (Figure 5) that separates the routes whose most important sections run through a siliceous terrain with its characteristic flora, which is widely used in popular veterinary medicine, especially in the provinces of Ciudad Real and Toledo, from the routes that run through the limestone terrain of Albacete and Cuenca, and link the Eastern Mancha and the “Serranía de Cuenca” with Andalusia and the Spanish Levant. The flora of calcareous territories differs to a large extent from the previous one and, therefore, provides its own medicinal resources.

Due to differences in substrate and climate, the plant species available along the routes in the western and eastern sectors differ (Table 5), resulting in the existence of characteristic groups in the repertoire of ingredients used. This confirms that one of the fundamental factors shaping the ethnoveterinary pharmacopeia is the local availability of resources.

**Table 5:** Different ingredients for the main groups of routes.

**Western transhumance routes**	**Common to both**	**Eastern transhumance routes**
*Cistus ladanifer* L.	*Daphne gnidium* L.	*Ruta angustifolia* Pers.
*Erophaca baetica* (L.)Boiss.	*Olea europaea* L.	*Stipa tenacissima* L.
*Ruta montana* (L.)L.	*Sambucus nigra* L.	*Marrubium vulgare* L.
*Rosmarinus officinalis* L.	*Nerium oleander* L.	*Sideritis tragoriganum* Lag. subsp. *mugronensis* (Borja) Obón & Rivera
*Mentha cervina* L.	*Plumbago europaea* L.	*Silene muscipula* L.
*Malva sylvestris* L.	*Vitis vinifera* L.	*Eryngium campestre* L.
*Mentha pulegium* L.	NaCl (Sodium Chloride)	*Nicotiana tabacum* L. (cultivated)
*Quercus rotundifolia* Lam.	*Juniperus oxycedrus* L. var. *oxycedrus*	*Pinus nigra* subsp. *salzmannii* (Dunal) Franco
*Drimia maritima* (L.) Stearn	*Ilex aquifolium* L.	*Lysimachia ephemerum L*.
*Sus domesticus* Erxleben, 1777	*Retama sphaerocarpa* (L.) Boiss.	*Homo sapiens* Linnaeus, 1758
*Bryonia cretica* subsp. *dioica* (Jacq.) Tutin	*Paeonia broteri* Boiss. & Reut.	*Ruta angustifolia* Pers.
*Lupinus angustifolius* L.	*Juniperus oxycedrus* var. *badia* H.Gay	
*Pistacia terebinthus* L.		
*Oenanthe crocata* L.		
*Verbascum rotundifolium* Ten.		
*Delphinium staphisagria* L.		
*Lavandula stoechas* L.		
*Conium maculatum* L.		
*Arbutus unedo* L.		
*Verbascum thapsus* L.		
*Vincetoxicum hirundinaria* Medik.		

*Among the 202 registered ingredients, only those that are characteristic within one of the three categories (Western, common and Eastern) are represented in the table, in decreasing order of number of records*.

There are very notable examples of how species are substituted, from east to west, depending on their availability; for example, among the species of rue, *Ruta montana* is predominant in westernmost routes, on siliceous grounds in many cases, while in the east, on routes that run through calcareous soils, it is replaced by *R. angustifolia* (Table 5). The medicinal use of the various species of rue has a long tradition (166).

These substitutions are also recorded in emic categories such as the vernacular names of plants: the plant called “arnica,” a name that in the Iberian Peninsula covers more than 30 different species of plants (167). In the case of the transhumance routes, the species named “arnica” in the western ones is *Achillea ageratum* L., and in the eastern ones, it is *Chiliadenus glutinosus* (L.) Fourr. Both species are used in decoction to wash wounds of transhumant cattle.

*Sideritis* species are characteristic of the eastern routes (Table 5), which is coherent with the geographical patterns previously recorded for their medicinal uses (168–170). In ethnoveterinary medicine, these are used in decoction, alone or mixed with *Salvia officinalis* subsp. *lavandulifolia* (Vahl) Gams (=*S. lavandulifolia* Vahl.).

These “Vías Pecuarias” are not only roads for transhumant cattle but also for people with ideas, customs, and knowledge that also travel along them, which are also exchanged between wintering pastures and summer pastures. As roads that they are, they connected distant areas hundreds of kilometers away and allowed for a process of cultural fusion and assimilation of knowledge and practices. Staying for months in the natural environment in two different areas allowed shepherds to familiarize with the vegetation and fauna of both areas. They mention similarities and differences. Wolf attacks were more frequent in Sierra Morena. The shepherds who did annual transhumance, like those of Tragacete (Cuenca), knew perfectly well the flora of both regions and their uses (52). They told us about the “abelfa” (*Nerium oleander*) and the “arrayán” (*Myrtus communis*) that grow abundantly in Sierra Morena and Los Pedroches Valley (171, 172) and about the wild olive wood with which they made the clappers there, while in Montes Universales they used the bush (*Buxus sempervirens* L.) for the same purposes. Some plants shared the same name, undoubtedly because of a similitude of properties. Thus, in Sierra Morena, the “garbancillo” (*Erophaca baetica*) is recognized for its toxicity, and another equally toxic plant, *Aconitum napellus*L., receives in Cuenca the same name of “garbancillo.” Livestock care and management also include these adaptive and nomadic knowledge and practices.

### Medicinal and Veterinary Value of Traditional Knowledge

The movement of cattle for almost a month, from the wintering areas to the summer pastures and vice versa twice a year, therefore, increases the risk of injuries and other ailments. For this reason, it was essential to carry a veterinary medicine cabinet in the herd, traditionally “miera” and little else, and currently other medicines but always supported at all times with biological resources for medicinal use (plants, fungi, and animals) and mineral materials that were available in each site. Undoubtedly, these routes come to represent a true corridor of traditional knowledge and cultural biodiversity.

In medicine for humans, there is fragmentary evidence that suggests a direct relationship between specific locally prevalent pathologies and elaboration of determined herbal mixtures to be consumed in the form of teas or decoctions, or as hydro-alcoholic extracts obtained by maceration. This connection is attributed to different active compounds provided by herbal ingredients (173–177).

The richness of this type of knowledge is linked to daily practice and significant reduction in the number of heads of transhumant cattle and, consequently, of shepherds, and break in the transmission of traditional knowledge among generations are leading to a considerable loss of ethno-veterinary knowledge. We must, here, mention that the three most relevant informants in this study were born between 1918 and 1924 and are deceased today.

The traditional knowledge collected in this study is not original or exclusive for the most part but is linked to the centuries-old European veterinary tradition. A good proof is that the vernacular names of the diseases appear collected in “albeitería” treatises, in some cases, since the Middle Ages (89–91, 106).

We must emphasize that the profile of the knowledge of the Spanish population in the rural areas is suffering in the twenty-first century a profound change in the sense of a deep loss of knowledge associated with a trivialization (58). This has led the Spanish authorities to start the Spanish catalog of traditional knowledge linked to biodiversity (178–182). However, the data collected in the villages of Albacete, Cuenca, Ciudad Real, and Toledo along the main transhumance routes conform a series of over 25 years from 1994 to 2021 that still reflect a rich and diversified local culture, little influenced by globalization. This explains the high proportion of local ingredients.

## Conclusion

We compiled an ethnoveterinary inventory with 202 toxic and/or medicinal taxa from 92 families, where 164 are plants, 19 are animals, seven are minerals, five are fungi, two are algae, three are synthetic organic chemicals, two are animals, and inorganic chemicals and bacteria are one each. Most are local wild species (79%), followed by cultivated plants (12.4%) and livestock (c. 3%), and only a few are minerals (3.5%), chemicals (1.5%), or imports (0.5%). The proportion of imported species differs significantly from that in ethnopharmacological studies on the same area.

Whole plant and aerial parts, blooming or not, are the most frequently used plant parts followed by bark. Lamiaceae stands out with great difference in terms of records and species used, followed by Fabaceae. Asteraceae follows in terms of number of species but is relegated to 12th place in terms of number of records. The overrepresentation of Thymelaeaceae in the interviews is underlined, which is due to the single species of this family that is mentioned, *Daphne gnidium*, and to its popularity as a veterinary remedy and as a toxic plant.

The complexity of the formulations recorded is variable but low, with a maximum of five ingredients. Most of these formulations have only been described in a single interview, which confirms the vestigial character of the information we have collected and the heterogeneity and adaptive value of the culture from which they come.

With regard to gender and the amount of information recorded in terms of, respectively, records and ingredients mentioned, it was, on average, similar for men and women.

The extension of the animal health system and the veterinary control of the cattle herd have relegated most of these practices. Brucellosis has been declared an eradicated disease in Castilla-La Mancha, thanks to vaccination campaigns. It would be necessary to speak more of substitution than of evolution and conclude that ethno-veterinary practices have remained as a very, very small complementary veterinary practice.

## Data Availability Statement

The original contributions presented in the study are included in the article/Supplementary Material, further inquiries can be directed to the corresponding author.

## Author Contributions

AVE, DR, CO, and JF were responsible for field work, interviews, collection and identification of samples, and ethnopharmacological analysis. AVA and JR-G were responsible of handling the herbarium and voucher specimens. AG-F and DR corrected the crude data in the data base. AVA and SR were responsible of administration and financial support. CO, CC, and JO carried out the bibliographic search. JP and DR are responsible of the multivariate analyses. DR is responsible for writing the manuscript. AVE and JF were responsible for photographs and FA for maps. All authors proof read manuscript and made contributions to the final version.

## Conflict of Interest

The authors declare that the research was conducted in the absence of any commercial or financial relationships that could be construed as a potential conflict of interest.

## Publisher's Note

All claims expressed in this article are solely those of the authors and do not necessarily represent those of their affiliated organizations, or those of the publisher, the editors and the reviewers. Any product that may be evaluated in this article, or claim that may be made by its manufacturer, is not guaranteed or endorsed by the publisher.

## Supplementary Material

Supplementary Table 1 Crude data with description of the formulas and simple remedies analyzed.

Supplementary Table 2 List of voucher specimens.
